# Randomized controlled trial reveals no benefit to a 3-month delay in COVID-19 mRNA booster vaccine

**DOI:** 10.1172/JCI181244

**Published:** 2024-07-11

**Authors:** Wen Shi Lee, Jennifer Audsley, Mai-Chi Trieu, Arnold Reynaldi, L. Carissa Aurelia, Palak H. Mehta, Joanne Patterson, Helen E. Kent, Julie Nguyen, Thakshila Amarasena, Robyn Esterbauer, Ebene R. Haycroft, Pradhipa Ramanathan, Miles P. Davenport, Timothy E. Schlub, Joseph Sasadeusz, Adam K. Wheatley, Amy W. Chung, Jennifer A. Juno, Kevin J. Selva, Stephen J. Kent

**Affiliations:** 1 Department of Microbiology and Immunology, Peter Doherty Institute for Infection and Immunity, University of Melbourne, Melbourne, Victoria, Australia.; 2 Department of Infectious Diseases, Peter Doherty Institute for Infection and Immunity, University of Melbourne and Royal Melbourne Hospital, Melbourne, Victoria, Australia.; 3 Influenza Centre, Department of Clinical Science, University of Bergen, Bergen, Norway.; 4 Kirby Institute, University of New South Wales, Kensington, New South Wales, Australia.; 5 Victorian Infectious Diseases Service, Royal Melbourne Hospital, Melbourne, Victoria, Australia.; 6 Sydney School of Public Health, Faculty of Medicine and Health, University of Sydney, Sydney, New South Wales, Australia.; 7 Melbourne Sexual Health Centre and Department of Infectious Diseases, Alfred Hospital and Central Clinical School, Monash University, Melbourne, Victoria, Australia.

**Keywords:** COVID-19, Vaccines, Adaptive immunity, Antigen, Immunoglobulins

## Abstract

**BACKGROUND:**

There is uncertainty about the timing of booster vaccination against COVID-19 in highly vaccinated populations during the present endemic phase of COVID-19. Studies focused on primary vaccination have previously suggested improved immunity with a longer interval between the first and second vaccine doses.

**METHODS:**

We conducted a randomized, controlled trial (November 2022–August 2023) and assigned 52 fully vaccinated adults to an immediate or a 3-month delayed bivalent Spikevax mRNA booster vaccine. Follow-up visits were completed for 48 participants (*n* = 24 per arm), with collection of saliva and plasma samples following each visit.

**RESULTS:**

The rise in neutralizing antibody responses to ancestral and Omicron strains were almost identical between the immediate and delayed vaccination arms. Analyses of plasma and salivary antibody responses (IgG, IgA), plasma antibody-dependent phagocytic activity, and the decay kinetics of antibody responses were similar between the 2 arms. Symptomatic and asymptomatic SARS-CoV-2 infections occurred in 49% (21 of 49) participants over the median 11.5 months of follow-up and were also similar between the 2 arms.

**CONCLUSIONS:**

Our data suggest that there was no benefit in delaying COVID-19 mRNA booster vaccination in preimmune populations during the present endemic phase of COVID-19.

**TRIAL REGISTRATION:**

Australian New Zealand Clinical Trials Registry number 12622000411741 (https://anzctr.org.au/Trial/Registration/TrialReview.aspx?ACTRN=12622000411741).

**FUNDING:**

National Health and Medical Research Council, Australia (program grant App1149990) and Medical Research Future Fund (App2005544).

## Abstract 2

Delay of the bivalent Spikevax mRNA booster by 3 months does not improve antibody immunity against SARS-CoV-2 in preimmune populations.

## Introduction

Ancestral spike–based COVID-19 vaccines have reduced effectiveness in preventing symptomatic Omicron variant infections due to progressive neutralizing antibody escape (1). As such, COVID-19 vaccines have been serially updated to include Omicron spike variants. Bivalent COVID-19 mRNA vaccines (BA.1, first approved in Australia in late 2022, followed by BA.5) are superior to ancestral monovalent vaccines at boosting Omicron neutralizing antibodies (2–4) and preventing hospitalization and severe disease (1, 5). Despite this, the bivalent mRNA boosters have shown only modest efficacy against infection with more recent XBB subvariants (6).

The durability of immunity afforded by bivalent booster vaccines and optimal timing for receiving boosters following the last vaccination or infection remain unclear. Guidance on the timing of repeated boosters varies widely. Early studies found that a longer interval between the first and second vaccine doses (8–16 weeks) elicited higher binding and neutralizing antibody titers compared with the standard 3- to 4-week interval (7–9), possibly due to improved maturation of antibody and B cell responses (10, 11). An extended interval between vaccination and infection also enhanced neutralizing antibody titers (12). However, a third mRNA vaccine dose equalized this response, resulting in similar neutralizing antibody titers in individuals who had short or long intervals between the first 2 doses (13).

The potential benefit of longer delays between subsequent boosters in highly vaccinated populations in the endemic phase of COVID-19 is currently unclear, with a fine balance between the potential for improved immunity with a longer duration between doses, the serial escape of Omicron strains leading to transient protective immunity, and vaccine fatigue within the population.

To determine whether there is an immunological benefit with a longer interval between the last vaccination or infection and the subsequent booster vaccination, we undertook an open-label, randomized, controlled trial in which we administered the Moderna BA.1 bivalent mRNA booster (mRNA-1273.214) upon recruitment (immediate arm) or 3 months after recruitment (delayed arm). We found that antibody-mediated immunity to circulating variants was not improved by delaying the booster.

## Results

### Study design.

**Figure 1:**
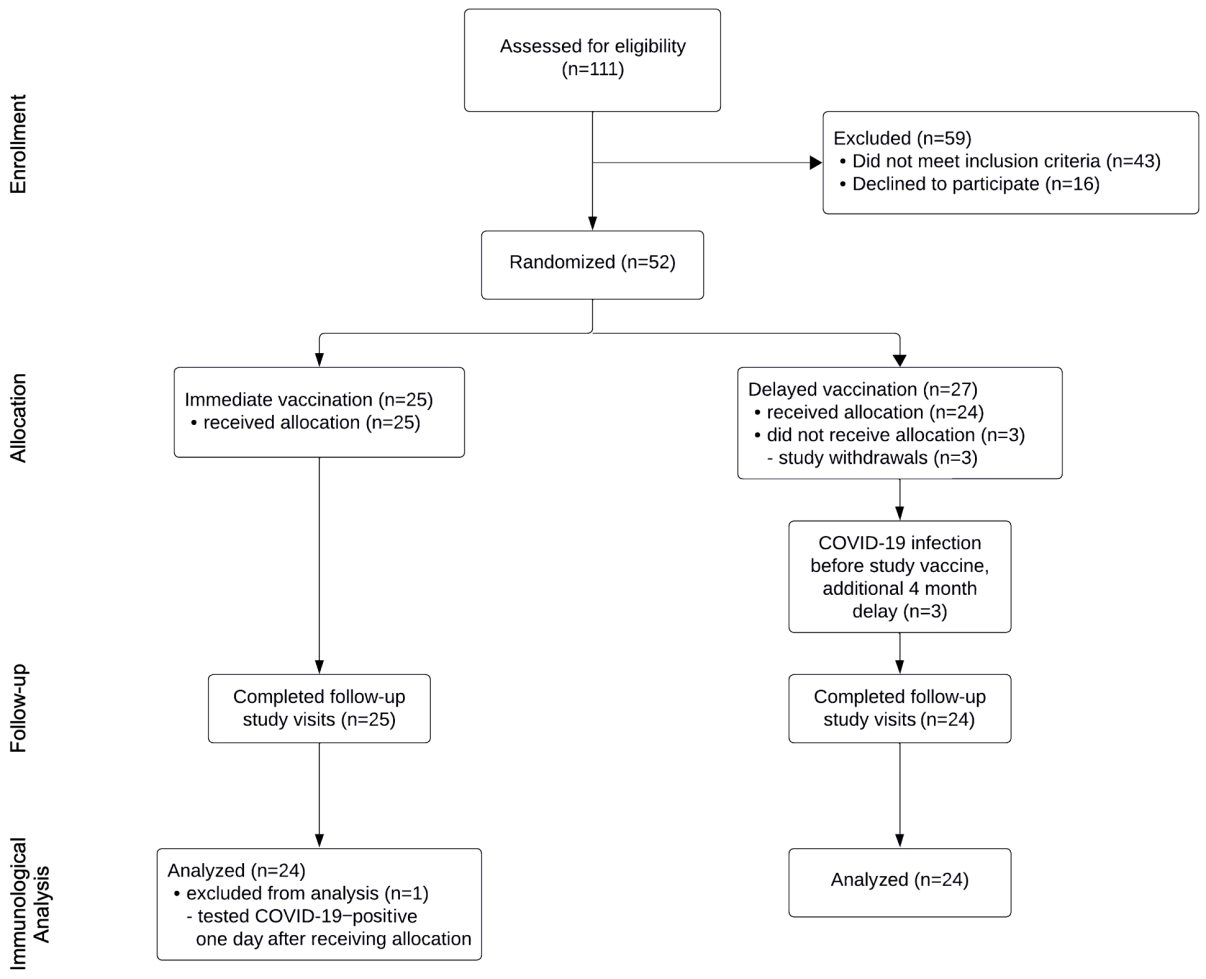
CONSORT flow diagram.

We recruited 52 adults over the period of September 11, 2022 to January 30, 2023, and 49 of these individuals completed follow-up (Figure 1) in this open-label randomized, controlled trial. The follow-up period was September 11, 2022 to December 2, 2023. Recruitment was stopped prior to reaching the predefined sample size in the immediate arm (*n* = 29 vs. *n* = 25 recruited) due to the withdrawal and replacement of the BA.1 bivalent vaccine with the BA.4/5 bivalent vaccine. Forty-eight participants (*n* = 24 randomized to the immediate vaccine arm; *n* = 24 randomized to the 3-month delay arm) were analyzed for immunologic outcomes, since 1 participant tested positive for SARS-CoV-2 1 day after receiving the vaccine (Supplemental Table 1; supplemental material available online with this article; https://doi.org/10.1172/JCI181244DS1). The relative immunogenicity of the Moderna Spikevax BA.1 bivalent vaccine booster in Australia was unknown at study initiation, and the primary outcome was a plasma neutralizing antibody titer of greater than 1:100 against Omicron BA.1 two weeks after vaccination in the immediate arm. Key additional endpoints included comparisons of antibody responses in plasma and saliva (mean titer of SARS-CoV-2 antibodies) between the 2 arms (Supplemental Figures 1 and 2), safety analyses (number of self-reported adverse events collected on days 3 and 7 after vaccination), and breakthrough COVID-19 infections during the study. Study participants were evenly matched for age, sex, the number of prior vaccinations, and the number of COVID-19 infections (Supplemental Table 1). The median time since the last vaccination or COVID-19 infection at enrollment was similar between the groups at 8.0 and 10.5 months for the immediate and delayed arms, respectively. Individuals in the delayed arm were given a booster vaccine a median of 3.1 months later than those in the immediate arm. Three participants randomized to the delayed arm acquired COVID-19 while waiting for vaccination and, as per protocol, waited 4 months after infection for their booster vaccine – two of these participants received the BA.4/5 bivalent Spikevax vaccine since the BA.1 bivalent vaccine had been withdrawn in the interim. One additional participant in the delayed arm also received the BA.4/5 bivalent Spikevax vaccine. There were 102 vaccine adverse events reported (Supplemental Table 2), with no statistically significant difference in reporting between the 2 arms. None of the adverse events were serious, and all were consistent with reactions reported previously (2).

### Bivalent vaccine boosts immune responses similarly in the immediate and delayed arms.

**Figure 2:**
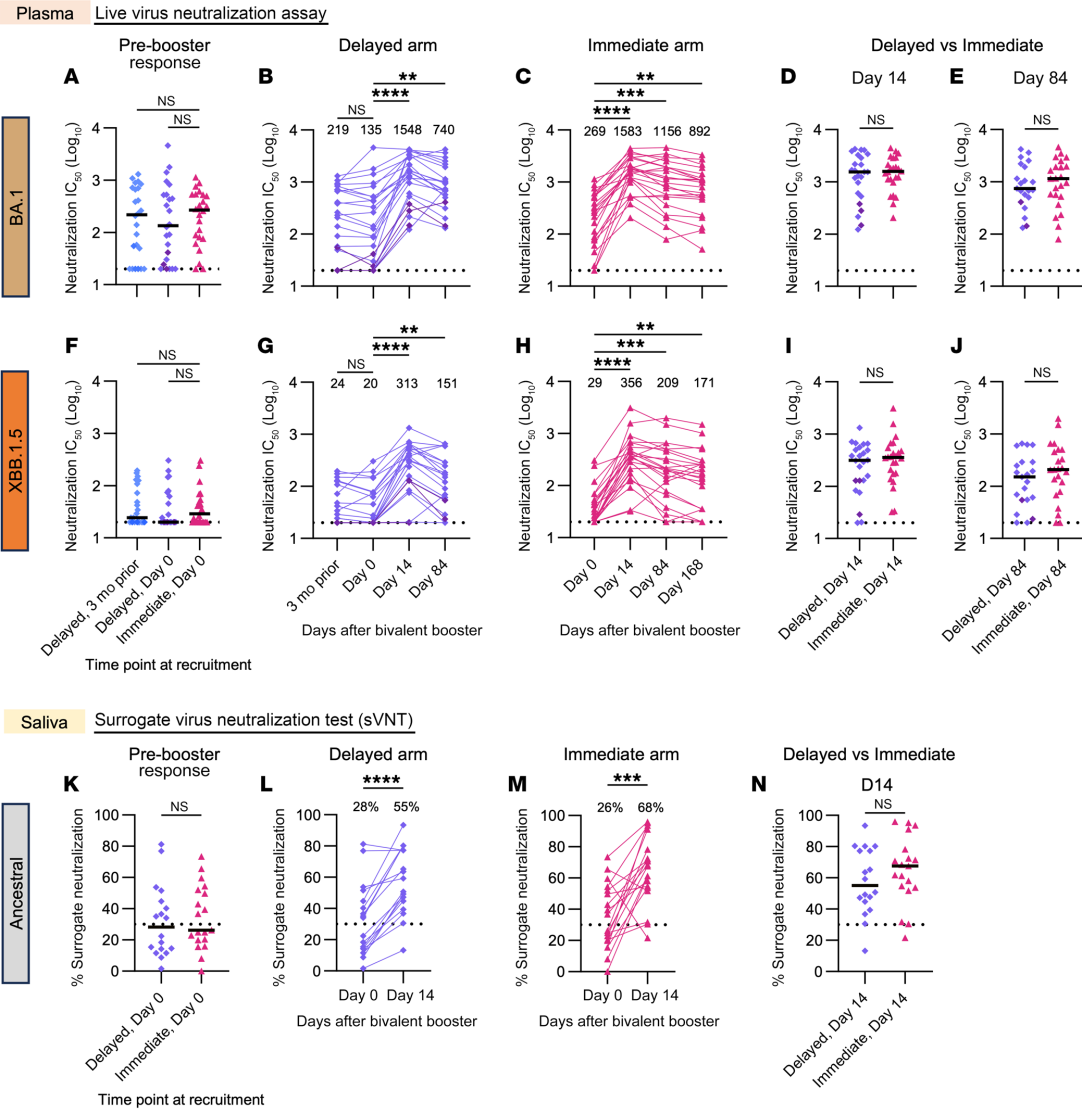
Neutralizing antibodies following bivalent mRNA booster vaccination. Plasma neutralizing activity was measured using a live virus neutralization assay against SARS-CoV-2 Omicron BA.1 (**A**–**E**) and XBB.1.5 (**F**–**J**) variants. Pre-booster (**A** and **F**) and post-booster day-14 (D14) (**D** and **I**) and day-84 (D84) (**E** and **J**) neutralizing antibody responses were compared between the delayed (blue/purple diamonds, *n* = 24) and immediate arms (pink triangles, *n* = 24) at the respective sampling time points. Line graphs describe the kinetics of plasma neutralization activity of the delayed (**B** and **G**) and immediate (**C** and **H**) arms after receiving the bivalent booster. Numbers above each time point describe the respective median neutralization IC_50_ against each viral variant. Dotted lines depict the detection threshold for the assay (neutralization IC_50_ = 20). Dark purple diamonds and lines show the antibody responses of the 3 individuals who received the BA.5 bivalent booster in the delayed arm of the study. Saliva neutralizing activity against ancestral SARS-CoV-2 was measured using the sVNT (Genscript). Pre-booster (**K**) and post-booster day-14 (**N**) neutralizing antibody responses were compared between the delayed (purple diamonds, *n* = 18) and immediate arms (pink triangles, *n* = 19), respectively. Line graphs describe the change in saliva neutralization activity following the bivalent booster (**L** and **M**). Numbers describe the percentage of surrogate neutralization observed at each time point. Dotted lines depict the sVNT cutoff for neutralizing activity (30%). Statistical significance was calculated between cohorts and time points using the 2-tailed Mann-Whitney *U* test or the Kruskal-Wallis test followed by Dunn’s multiple-comparison test. Paired saliva analysis (day 0 vs. day 14) was performed using the Wilcoxon matched-pairs, signed-rank test. Experiments were performed in duplicate. Graphs are displayed as the median, and where significant, *P* values are reported (**P ≤ 0.01, ***P ≤ 0.001, and ****P ≤ 0.0001).

Neutralizing antibodies are a key correlate of protective immunity against COVID-19 (14). Plasma neutralizing titers against BA.1 (in the booster) and XBB.1.5 (a dominant circulating Omicron strain during the study) were relatively low prior to vaccination in both groups (median IC_50_ = 219 and 269 for delayed and immediate arms for BA.1, respectively; IC_50_ = 24 and 29, respectively, for XBB.1.5 in a live virus neutralization test; Figure 2, A–C and F–H) despite a median of 3 prior COVID-19 vaccinations and 69.4% having at least 1 previous self-reported COVID-19 infection. For the delayed arm, BA.1 and XBB.1.5 neutralizing titers were similar from the study recruitment date (3 months before the booster) to the day of vaccination (day 0) (Figure 2, B and G). After receiving the booster, all immediate arm participants achieved an Omicron BA.1 neutralization titer of greater than 1:100 within 2 weeks (*P* ≤ 0.0001), meeting the study primary endpoint (Figure 2C). Neutralizing titers at day 14 after the booster were almost identical between the immediate and delayed arms to both Omicron BA.1 and XBB.1.5 (Figure 2, D and I), reaching median IC_50_ titers of 1,548 and 1,583 for BA.1, and 313 and 356 for XBB.1.5 in the delayed and immediate arms, respectively. At post-vaccination day 84, neutralizing titers decayed –1.4-fold to 2.1-fold from day 14 but remained similar between both arms (Figure 2, E and J).

Neutralizing activity against ancestral and XBB.1.5 strains across all sampled time points from both arms was also analyzed using a surrogate bead-based spike-ACE2 inhibition assay (Supplemental Figure 3, A–G). The percentage of inhibition against both ancestral and XBB.1.5 spike proteins peaked at similar levels at day 14 for both arms and gradually decayed over time.

Since antibodies in the upper airways may be important for preventing SARS-CoV-2 infection (15), we measured neutralizing antibody responses in saliva using an ELISA-based surrogate virus neutralization test (sVNT) (16) (Supplemental Figure 3, H–J). Salivary neutralizing antibodies against the ancestral strain were boosted in most participants at day 14 (*P* ≤ 0.001) and were similar in both the immediate and delayed arms (Figure 2, K–N).

Spike-specific T cells were recently implicated to be a predictor of protection against symptomatic infection in vaccinated children (17). In addition to quantifying the serological response to booster vaccination, we assessed the frequency of spike-specific CD4^+^ and CD8^+^ T cells at days 0 and 7 after vaccination in a subset of the cohort (Supplemental Figure 4A). Immunization drove a significant expansion of spike-specific memory for both CD4^+^ and CD8^+^ T cells, as measured by production of IFN-γ, IL-2, and/or TNF (Supplemental Figure 4, B and C; *P* = 0.007 for CD4^+^ memory T cells [Tmem], *P* = 0.016 for CD8^+^ Tmem). We did not detect any substantial spike-specific circulating T follicular helper cell (cTFH) responses, probably because of poor cytokine production by cTfh cells relative to other T cell subsets (18) (Supplemental Figure 4B). Spike-specific T cell frequencies at day 7 were comparable between the immediate and delayed vaccination arms for both CD4^+^ and CD8^+^ T cell populations (Supplemental Figure 4, D and E, and Supplemental Table 3), consistent with the serological data.

### Decay kinetics of vaccine-induced antibodies.

**Figure 3:**
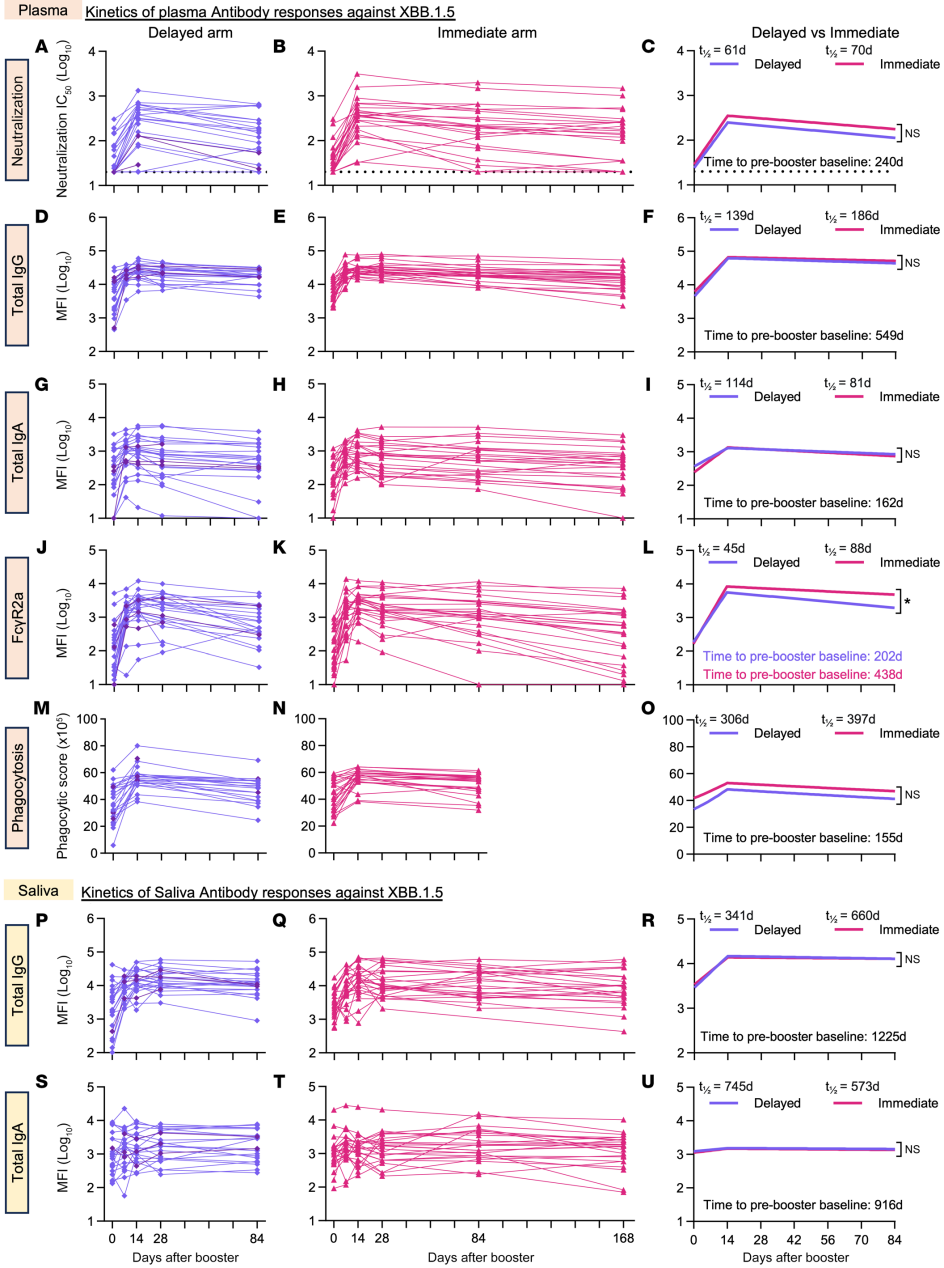
Antibody kinetics following bivalent mRNA booster vaccination. Kinetics of plasma (**A**–**O**) and saliva (**P**–**U**) antibody responses against the SARS-CoV-2 variant Omicron XBB.1.5. Line graphs depict the plasma neutralization responses in the delayed (purple diamonds, *n* = 24) (**A**) and immediate (pink triangles, *n* = 24) (**B**) arms as previously described in Figure 2, G and H. Line graphs also illustrate the rise and decay of plasma total IgG levels (**D** and **E**), total IgA responses (**G** and **H**), Fc-γR2a binding (**J** and **K**), and antibody-dependent phagocytic activity (**M** and **N**), as well as salivary total IgG levels (**P** and **Q**) and total IgA (**S** and **T**) responses in the delayed (purple diamonds, *n* = 24) (**D**, **G**, **J**, **M**, **P**, and **S**) and immediate (pink triangles, *n* = 24) (**E**, **H**, **K**, **N**, **Q**, and **T**) arms, respectively. Dark purple diamonds and lines show the antibody responses of the 3 individuals who received the BA.5 bivalent booster in the delayed arm of the study. Modeled decay slopes (**C**, **F**, **I**, **L**, **O**, **R**, and **U**) describe the half-life and time taken for the respective antibody responses to return to pre-booster baseline levels. Statistical significance was calculated between cohorts using the likelihood ratio test, and where significant, *P* values are reported (*P ≤ 0.05). Experiments were performed in duplicate.

Beyond peak antibody titers following vaccination, an important parameter of vaccine-induced antibodies is how fast they decay, leaving participants vulnerable to breakthrough infection (19). We analyzed differences in the decay kinetics of various antibody parameters across the immediate and delayed vaccination arms. Here, we studied not only plasma neutralizing antibody responses (Figure 3, A–C), but also total IgG and IgA levels in plasma (Figure 3, D–F and G–I) and saliva, respectively (Figure 3, P–R and S–U). Furthermore, as Fc-effector functions have been implicated in assisting antibody-mediated immunity to SARS-CoV-2 (20, 21), we also examined Fc-γ receptor 2a (FcγR2a) engagement and antibody-dependent cellular phagocytosis (ADCP) in plasma (Figure 3, J–O, and Supplemental Figure 5, A–E). While we focused the decay analyses on antibody responses to Omicron XBB.1.5 (Figure 3, A–U), as this was a major circulating strain during our study, we also examined total IgG and IgA and FcγR2a binding responses in plasma (Supplemental Figures 6, 8, 9, 11, and 13) and saliva (Supplemental Figures 7, 8, 10, 12, 13), respectively, against ancestral, Omicron BA.1, and Omicron BA.5 strains.

The decay kinetics of plasma neutralizing antibodies (Figure 3, A–C), as well as total IgG and IgA against XBB.1.5 spike in plasma (Figure 3, D–I) and saliva (Figure 3, P–U), respectively, were very similar between the immediate and delayed arms out to 84 days after the booster. Of note, spike-specific salivary IgA responses were not induced by the vaccine, consistent with the known poor mucosal immunity induced by intramuscular vaccines (Figure 3, S and T, and Supplemental Figure 10) (22, 23). FcγR2a-binding antibodies against spike in plasma were elicited by the vaccine and had a modestly faster decay rate in the delayed arm (*t_1/2_* = 45 vs. 88 days, *P* ≤ 0.05; Figure 3, J–L, and Supplemental Figure 10). However, this difference diminished when we compared Fc effector responses of plasma antibodies using a cell-based phagocytosis assay (ADCP) (Figure 3, M–O). Overall, our results suggest that delaying vaccination in the context of our study had no substantial benefit in terms of preserving long-term antibody immunity.

We also modeled the time required for the various XBB.1.5 antibody responses to decrease to pre-booster levels (Figure 3, C, F, I, L, O, R, and U). Plasma neutralizing titers against XBB.1.5 took an average of 240 days to decay to baseline levels. Saliva IgG took the longest time to decay (1,225 days), whereas plasma IgA took the shortest time (162 days).

### COVID-19 infections during the study.

**Figure 4:**
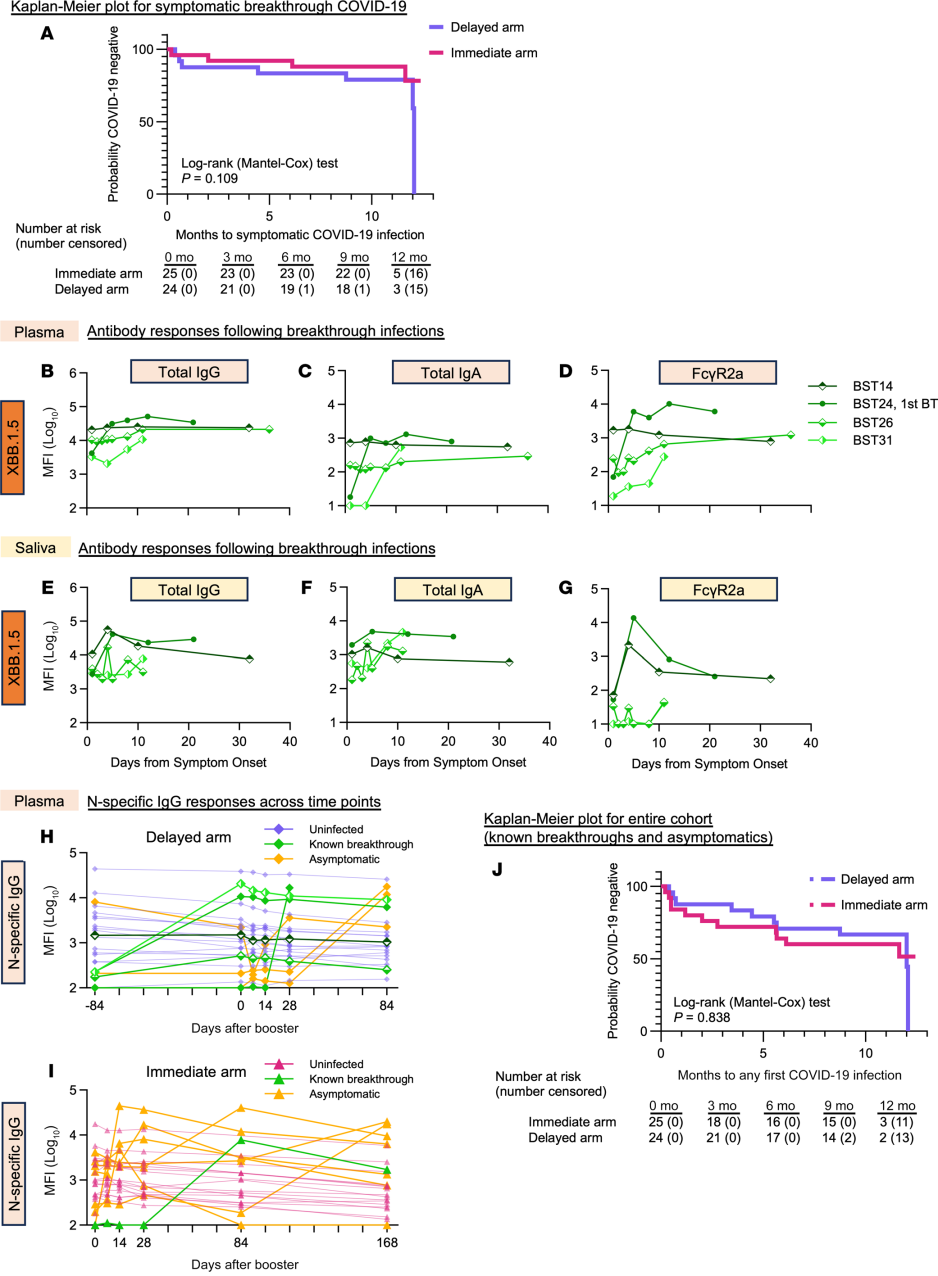
Breakthrough COVID-19. Kaplan-Meier probability of remaining negative for symptomatic COVID-19 during the study in the delayed (purple) and immediate (pink) arms (**A**). Analysis includes all first on-study COVID-19 symptomatic infections (pre- and post-study vaccination, self-reported). The probability for the delayed arm reaches zero because the final 3 delayed arm participants were positive/censored just after 12 months, whereas there were 5 final immediate arm participants who remained at risk. The numbers below the graph show the remaining number of participants at risk (number censored) during the study at baseline (0 mo), month 3 (3 mo), month 6 (6 mo), month 9 (9 mo), and month 12 (12 mo). Statistical significance between survival curves was calculated by log-rank Mantel-Cox test. Line graphs show the plasma (**B**–**D**) and salivary (**E**–**G**) antibody responses against Omicron XBB.1.5 from 4 representative individuals (green) with COVID-19 breakthrough infections (rapid antigen test–positive [RAT-positive]). Total IgG (**B** and **E**), total IgA responses (**C** and **F**), and Fc-γR2a binding (**D** and **G**) against Omicron XBB.1.5 are shown following symptom onset. Line graphs also depict the kinetics of N-specific IgG for both the delayed (purple diamonds) (**H**) and immediate (pink triangles) (**I**) arms across sampling time points, highlighting individuals with known symptomatic (RAT-positive; green) and asymptomatic (>4-fold rise in N-specific IgG from the previous time point; yellow) breakthrough infections. Experiments were performed in duplicate. (**J**) Kaplan-Meier plot showing the probability of remaining COVID-19 negative during the study in the delayed (purple) and immediate (pink) arms. Analysis includes all first on-study COVID-19 infections (pre- and post-study vaccination, self-reported, and asymptomatic laboratory diagnosis). The probability for the delayed arm reaches zero because the final 2 delayed arm participants were positive/censored just after 12 months, whereas there were 3 final immediate arm participants who remained at risk (log-rank *P* = 0.838, by Mantel-Cox test).

Australia has experienced multiple waves of COVID-19, including during the current study. Although not powered for efficacy, we documented symptomatic COVID-19 infections over the course of follow-up. We identified 14 symptomatic infections out to a maximum follow-up of 12.4 months (Supplemental Table 4). This included 2 participants who reported 2 symptomatic infections (1 participant in each arm). The symptomatic infections were evenly distributed between the immediate and delayed arms, with similar Kaplan-Meier lines (Figure 4A, Log-rank Mantel-Cox test; *P* = 0.109). The apparent reduction in COVID-free survival in the delayed arm was because the last participant in the follow-up contracted COVID-19. All documented infections were mild in severity, consistent with multiple prior vaccinations.

Analyses of serial immune responses following breakthrough COVID-19 have been informative regarding the recall of immunity that helps control infection (24–26). Little is known about serial salivary antibody responses following breakthrough COVID-19 with recent Omicron strains. We were able to obtain nasal swab samples for 4 study participants with breakthrough COVID-19 during the trial and found that 3 of 4of these individuals acquired the XBF strain (viral sequencing was unsuccessful in the last nasal swab). We also obtained additional serial saliva and blood samples and analyzed antibody responses (Figure 4, B–G, and Supplemental Figure 14). We detected transient rises in XBB.1.5-specific total IgG and IgA and FcγR2a binding responses in both plasma and saliva in 3 of the 4 study participants (Figure 4, B–G), confirming that breakthrough COVID-19 can boost mucosal immunity.

Since asymptomatic SARS-CoV-2 infections are also common, we analyzed non-vaccine- elicited antibodies against the N protein. We identified 10 participants without symptomatic COVID-19 during our study, who had a clear and sustained rise in N antibodies (>4-fold increase over the previous sampling time point; Figure 4, H and I) and a rise in XBB.1.5 neutralization titers. Combined cases of symptomatic and asymptomatic infection were evenly divided between the arms and similar over time (Figure 4J, log-rank Mantel-Cox test; *P* = 0.838).

## Discussion

The timing of SARS-CoV-2 booster vaccination is contentious in highly vaccinated populations in the present endemic phase of COVID-19, with waning immunity, changing escape profiles of new variants, and booster fatigue all factors to consider. We randomized healthy adults to receive an immediate or 3-month-delayed COVID-19 booster vaccine. The booster improved antibody and T cell immunity in all participants. We found no difference in booster-induced antibody-based immunity to either ancestral, vaccine (BA.1), or circulating strains of SARS-CoV-2 (XBB.1.5) between the immediate and delayed arms. Furthermore, we noted no improvement in the decay kinetics of spike-specific antibodies over the subsequent 12 weeks in the delayed arm, suggesting no longer-term benefit from delaying vaccination. Remarkably, over 40% of the participants (21 of 49) completing the study had symptomatic or asymptomatic COVID-19 during the mean 11.5-month study follow-up period, but the rates of infection were similar in both arms. Taken together, our results suggest that there was no substantial benefit in delaying booster vaccination to improve antibody-based immunity to SARS-CoV-2.

The changing landscape of SARS-CoV-2 Omicron variants is a major factor driving poor immunity and breakthrough COVID-19 infections. The levels of neutralizing antibodies against Omicron XBB.1.5 (which was a common circulating strain during our study) were low before the booster (median IC_50_ = 24 and 29, respectively, with 75% being <1:100). XBB.1.5 titers reached a median of 346 across the whole cohort 2 weeks after vaccination, consistent with a previous study showing that BA.1 bivalent vaccines boosted neutralizing titers against XBB.1.5 (27), despite the poor effectiveness against symptomatic XBB.1.5 infection (6). XBB.1.5 titers waned to a median of 186 by day 84 and were estimated to return to the low pre-booster baseline levels by an average of 240 days after receiving the booster. This illustrates the relatively short-lived effect of current mRNA booster vaccines.

Although the BA.1 bivalent vaccine we studied has been superseded with a XBB.1.5 monovalent vaccine (28), recent dominant Omicron strains such as JN.1 have continued to escape neutralizing antibody responses (29). Maintaining high levels of neutralizing antibodies against circulating and emerging variants with the current process of updating vaccines is inefficient, resulting in increasing cases of COVID-19 breakthrough infections, as we observed. Nonetheless, delaying booster vaccination with the hope of improving the peak or durability of antibody immunity during the present endemic phase of COVID-19 does not work, nor does it prevent COVID-19. There is a need for vaccines that elicit broader and more durable protective immunity against SARS-CoV-2.

Our study has limitations. First, our study had 24 participants per arm, who were analyzed for antibody immunity owing to intercurrent COVID-19 infections and the update of the bivalent vaccine. Although participant numbers were adequate for most analyses, our ability to detect small differences in the peak or waning of antibodies between the 2 arms was less robust. However, the virtually identical levels of neutralizing antibody responses, confirmed with multiple other analyses of antibody responses, suggests that any real difference between immediate or delayed vaccination would be very small and of doubtful clinical significance. Second, there were many intercurrent asymptomatic and symptomatic SARS-CoV-2 infections and presumably many more exposures to SARS-CoV-2 that did not lead to overt infections during our study. These COVID-19 breakthrough infections also modulate antibody responses (24–26), as documented here in several cases (Figure 4, B–G, and Supplemental Figure 14). Although these infections and exposures could confound some of our antibody analyses, the infections were evenly distributed between the 2 arms and unavoidable, given circulating SARS-CoV-2 levels during our study. Third, our participants had an average of 3 prior vaccinations and an average time of 9.4 months from prior vaccination or COVID-19 infection. There might be scenarios with fewer prior vaccinations and/or COVID-19 infections, or different timing of booster vaccination that could reveal differences in immediate or delayed vaccination. Too short a time between a COVID-19 infection and a booster vaccine has been shown to be suboptimal (30). However, pre-booster neutralizing antibodies against the circulating XBB.1.5 variant were low in our study, and a significant proportion of our study population acquired SARS-CoV-2 infection during our trial. This suggests that we studied a relevant population in efforts to improve immunity to and protection from infection. Fourth, we studied a group of healthy adults who were younger than 65 years of age, while immunocompromised or elderly groups — key target groups for vaccination — may respond differently and have a larger benefit from more frequent booster vaccinations (31). Last, our assays to date have been largely focused on antibody immunity, while cellular immunity could theoretically be modulated to a greater degree by vaccination timing and potentially play an important role in long-term immunity (32). Nevertheless, neutralizing antibodies have emerged as a robust correlate of immunity to SARS-CoV-2 and guide most vaccine recommendations (14, 19).

In summary, this randomized, controlled trial of highly vaccinated healthy adults during the present endemic phase of COVID-19 showed no improvement in the induction of protective antibodies against SARS-CoV-2 by delaying booster vaccination 3 months. Regular booster SARS-CoV-2 vaccinations are supported by the findings of this study.

## Methods

### Sex as a biological variable.

Male and female participants were enrolled in this study, which was open to all sexes. Randomization included matching for sex.

### Study participants.

Adults (18–65 years of age) who had received 2–3 doses of COVID-19 vaccines at least 4 months prior were eligible to participate in this study. Exclusion criteria included prior COVID-19 infection in the previous 4 months, immunosuppression, and previous significant adverse events related to COVID-19 vaccines. A SARS-CoV-2 Omicron blood neutralizing titer above 1:100 in greater than 90% of participants was considered a successful outcome, since this level is predicted to be reliably protective against the Omicron strain. On this basis, power calculations were carried out with G*Power, version 3.1.9.7, using a 1-tailed exact generic binomial test. Twenty-nine participants in the immediate vaccination group were estimated to be required for a greater than 90% proportion of participants with a neutralization titer of over 1:100. Dynamic (adaptive) randomization with minimization to promote a balance in age, sex, and timing of the initial vaccines was used to allocate participants to either interventional group. Age was stratified by 10-year intervals and time since the second vaccine by monthly intervals, using equal weighting of covariate factors. This was achieved using R, a language and environment for statistical computing and the library Minirand, function Minirand, using equal weighting of covariate factors and a high probability of assignment = 0.90. Participants were recruited in Melbourne, Australia, and were randomized to receive a Moderna BA.1 bivalent mRNA vaccine booster dose (0.5 mL) administered intramuscularly upon enrollment (immediate arm) or 3 months later (delayed arm). Most participants received the Moderna BA.1 bivalent vaccine, however, during the study the Moderna BA.4/5 bivalent vaccine replaced the BA.1 formulation, and 3 participants received the BA.4/5 vaccine. Participants were randomized to the 2 study arms and matched for age (10-year intervals), sex (male, female), and timing of the last COVID-19 vaccine dose (2-month intervals, from a minimum of 4 months). The study was open labeled.

Serial blood plasma samples and saliva samples (SalivaBio, Salimetrics) were collected and stored at –80°C. Saliva samples from individuals in the delayed and immediate arms had comparable levels of total secretory IgA between the respective time points (Supplemental Figure 1). PBMCs were isolated from whole blood by Ficoll-Paque separation and cryopreserved in 10% DMSO and 90% FCS.

### Variant spike multiplex bead assay.

SARS-CoV-2–specific total IgG, IgA, and FcγR2a dimer (Bruce Wines, Burnet Institute) engagement in plasma (1:25,600, 1:6,400, 1:6,400) and saliva (1:50, 1:50, 1:12.5) from the booster cohort were assessed using a customized multiplex bead-based array consisting of ancestral and Omicron spike trimers (BA.1, BA.5, XBB.1.5, Sino Biological), as previously described (26) (Supplemental Figure 2). SARS-CoV-2 nucleocapsid (N) protein was included to screen for asymptomatic infections. SIVgp120, H1Cal2009 (Sino Biological), and tetanus toxoid (MilliporeSigma) were included as controls. Briefly, spike-coupled beads were first incubated with samples overnight at 4°C and then washed and incubated with biotinylated detectors (isotype detection antibodies, MabTech; FcγR2a dimers) for 2 hours at room temperature (RT). After washing, the beads were incubated with streptavidin-R-phycoerythrin (Thermo Fisher Scientific) for 2 hours at RT. The beads were washed again and read on the Intelliflex (Luminex). The assays were repeated in duplicate.

### Virus neutralization assay.

A plasma live virus neutralization assay with viability dye readout was performed against Omicron BA.1 and XBB.1.5 viruses as previously described (33). Infectivity of virus stocks was determined by titration on HAT-24 cells (a clone of transduced HEK293T cells stably expressing human ACE2 and TMPRSS2) (34). Virus stocks were titrated in quintuplicate in 3 independent experiments to obtain mean 50% infectious dose (ID_50_) values.

To determine serum neutralization activity, heat-inactivated plasma samples were diluted 3-fold (1:20 to 1:43,740) in duplicate and incubated with SARS-CoV-2 virus at a final concentration of 2 × ID_50_ at 37°C for 1 hour. Next, 40,000 freshly trypsinized HAT-24 cells in DMEM with 5% FCS were added and incubated at 37°C. “Cells only” and “virus + cells” controls were included to represent 0% and 100% infectivity, respectively. After 48 hours, 10 μL alamarBlue Cell Viability Reagent (Thermo Fisher Scientific) was added to each well and incubated at 37°C for 1 hour. The reaction was then stopped with 1% SDS and read on a FLUOstar Omega plate reader. The relative fluorescence units (RFU) measured were used to calculate the neutralization percentage with the following formula: (sample – virus + cells)/(cells only – virus + cells) × 100. Fifty percent inhibitory concentration (IC_50_) values were determined using 4-parameter, nonlinear regression in GraphPad Prism with the curve fit constrained to have a minimum of 0% and a maximum of 100% neutralization.

### sVNT.

Neutralizing activity of plasma (final dilutions, 1:6,400) were also assessed using an adapted surrogate spike-ACE2 inhibition assay (35) (Supplemental Figure 3, A–G). Briefly, ancestral or Omicron XBB.1.5 variant S1-coupled beads were incubated with diluted plasma overnight at 4°C. Avi-tagged biotinylated ACE2 (provided by Nicholas Gherardin, University of Melbourne, Melbourne, Australia) was added, and the beads were incubated for 1 hour at RT. After washing, the beads were incubated with streptavidin-PE for 1 hour at RT, and then R-phycoerythrin biotin-XX conjugate (Thermo Fisher Scientific) was added for a 1-hour incubation at RT. The beads were washed and read on the Intelliflex. The assays were repeated in duplicate. Saliva neutralizing activity against the ancestral virus in saliva (final dilutions, 1:2) and plasma (final dilutions, 1:200) samples were measured using the sVNT kit (GenScript cPass) as per the manufacturer’s directions. Readings above the recommended 30% cutoff were positive for neutralizing activity (Figure 3, H–J).

### Bead-based THP-1 ADCP assay.

A bead-based ADCP assay was performed as previously described (Supplemental Figure 5A) (36). Briefly, SARS-CoV-2 XBB.1.5 spike trimer (Sino Biological) was biotinylated and coupled to 1 μM fluorescent NeutrAvidin Fluospheres beads (Invitrogen, Thermo Fisher Scientific) overnight at 4°C. Antigen-coated beads were washed and diluted in 1% BSA and PBS and incubated with plasma (final dilutions, 1:1,600; Supplemental Figure 5B) for 2 hours at 37°C in a 96-well, U-bottomed plate. THP-1 monocytes (100,000/well) were added to opsonized beads and incubated for 16 hours under cell culture conditions. THP-1 monocytes were then fixed and acquired by flow cytometry on a BD LSR Fortessa with a high-throughput sampler. The data were analyzed using FlowJo 10.9.0 (see Supplemental Figure 5A for the gating strategy), and a phagocytosis score was calculated as previously described using the formula: percentage of bead-positive cells × MFI.

### Spike-specific T cell assays.

Cryopreserved PBMCs were thawed and rested for 4 hours in RPMI-1640 supplemented with 10% FCS and penicillin-streptomycin (RF10). PBMCs (2 × 10^6^) were seeded in each well in a 96-well, U-bottomed plate and stimulated with 1 μg/mL of a peptide pool covering the spike protein (PepTivator SARS-CoV-2 Prot_S Complete) or an equivalent volume of vehicle control (sterile H_2_O). After 1 hour, brefeldin A (Golgi Plug, BD Biosciences) was added to the cell culture. PBMCs were cultured for a total of 16 hours before being washed with PBS. Cells were stained with live/dead (Invitrogen, Thermo Fisher Scientific) for 3 minutes at RT and then incubated with the surface antibody cocktail for 30 minutes at 4°C. The surface antibody cocktail included the following: CD20 BV510, 2H7; CD3 BUV395, SK7; CD27 BUV737, L128; CXCR5 BB515, RF8B2 (all from BD Biosciences); CD4 BV605, RPA-T4; CD8 BV650, RPA-T8; and CD45RA PerCP-Cy5.5, HI100 (all from BioLegend). After fixation and permeabilization (BD CytoFix/CytoPerm) for 20 minutes at 4°C, cells were incubated with the intracellular antibody cocktail (IFN-γ APC, B27; TNF BV421, Mab11; IL-2 PE, MQ1-17H12; all from BioLegend). Cells were washed in Perm/Wash buffer, resuspended in PBS plus 1%FCS, and acquired on a BD LSR Fortessa.

### Modeling.

A piece-wise model was used to estimate the growth and decay rate of various immune responses following vaccination. The model of the immune response for participant *i* at time *y_i_* can be written as:

 (Equation 1)
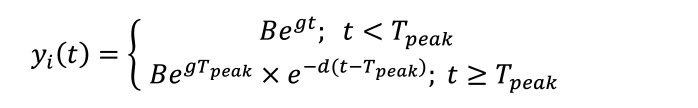


The model has 4 parameters: *B*, *g*, *T_peak_*, and *d*. We assumed a constant baseline value for the immune response before vaccination. The immune response will grow at a rate of *g* until *T_peak_*. From *T_peak_*, the immune response will decay at a rate of *d*. For each participant, the parameters were taken from a normal distribution, with each parameter having its own mean (fixed effect). A diagonal random effect structure was used, whereby we assumed there was no correlation within the random effects. The model was fitted to the log-transformed data values, with a constant error model distributed around zero with a SD. We also censored the data from below (left-censoring) if they were less than the threshold for detection. Model fitting was performed using Monolix2023R1.

### Statistics.

Statistical analysis was performed with GraphPad Prism 10.2.0 (GraphPad Software). Antibody responses among cohorts/time points/variants are presented as medians and compared using a 2-tailed Mann-Whitney *U* test, a Kruskal-Wallis test followed by Dunn’s test for multiple comparisons, a Friedman test followed by Dunn’s test for multiple comparisons, or a Wilcoxon matched-pairs, signed-rank test where appropriate. *P* values of 0.05 or less were considered significant.

### Study approval.

The study was approved by ethics committees at the Royal Melbourne Hospital (study no. 2021/272) and the University of Melbourne (approval nos. 13793 and 23497). Written informed consent was obtained from all participants prior to enrollment in the study. This study was registered with the Australian New Zealand Clinical Trials Registry (anzctr.org.au, no. 12622000411741).

### Data availability.

All the data and methods are presented in the manuscript or in the supplemental materials. All individual values for figures are available in the Supporting Data Values file.

## Author contributions

SJK conceived and designed the study. JS, JP, and HEK recruited the study participants. TES generated the random allocation sequence and assigned participants to the interventions. KJS, WSL, LCA, PHM, JA, MCT, JP, HEK, JN, TA, RE, ERH, PR, TES, JS, AWC, AKW, SJK, and JAJ were responsible for the acquisition of data. KJS, WSL, JA, AR, MPD, JAJ, and SJK performed the analyses and interpreted the results. SJK, KJS, WSL, and JA wrote the first draft of the manuscript. All authors critically revised the report and approved the final version. The order of co–first authors’ names was assigned on the basis of their experimental and editorial contributions to this study.

## Supplementary Material

Supplemental data

ICMJE disclosure forms

Supporting data values

## Version 1 07/11/2024

In-Press Preview

## Version 2 09/03/2024

Electronic publication
