# Supplementary material for: Randomized controlled trial reveals no benefit to a 3-month delay in COVID-19 mRNA booster vaccine
Source: J Clin Invest. 2024 Jul 11;134(17):e181244. doi: 10.1172/JCI181244 (PMC11364387; doi:10.1172/JCI181244)
Supplement: Supplemental data [file jci-134-181244-s172.pdf]

Saliva Saliva secretory IgA assay

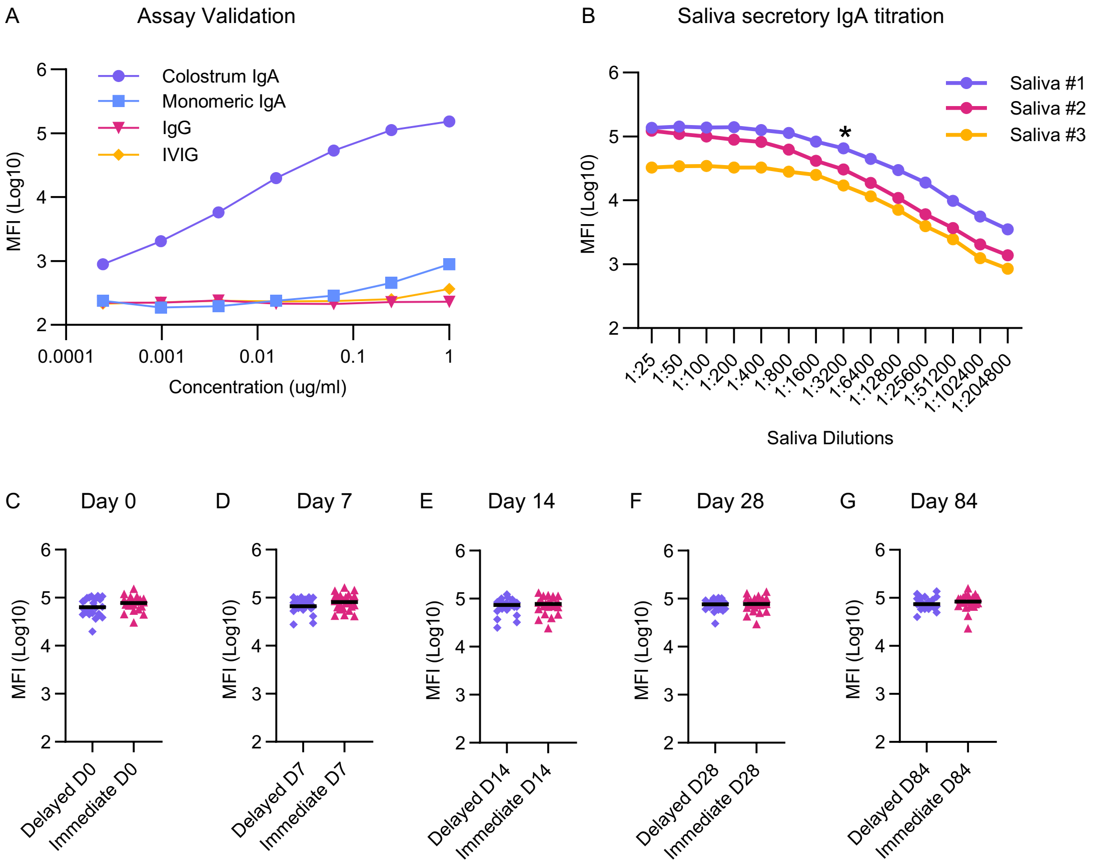

Supplementary Figure 1: IgA assay validation and titrations

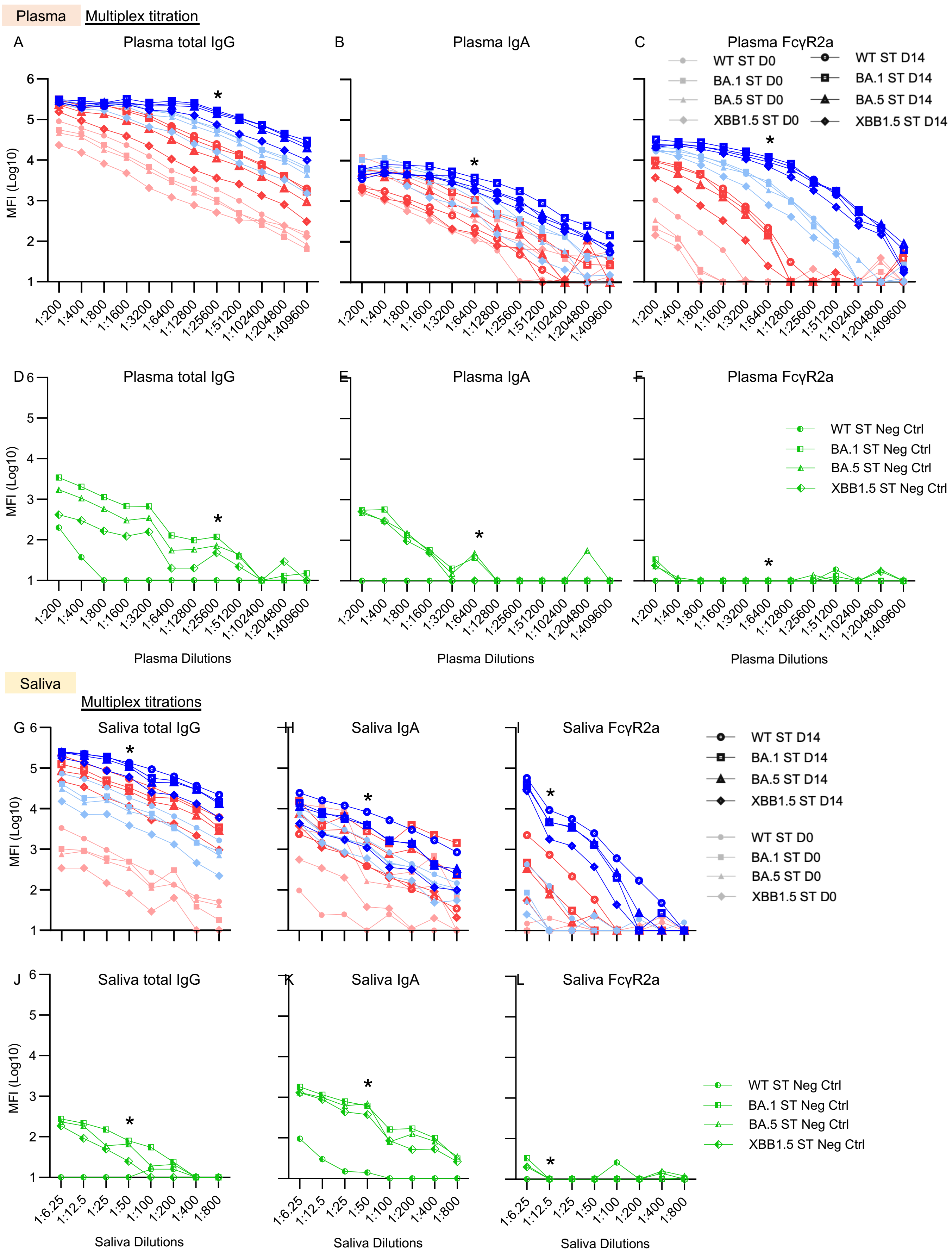

Supplementary Figure 2: Multiplex assay validations

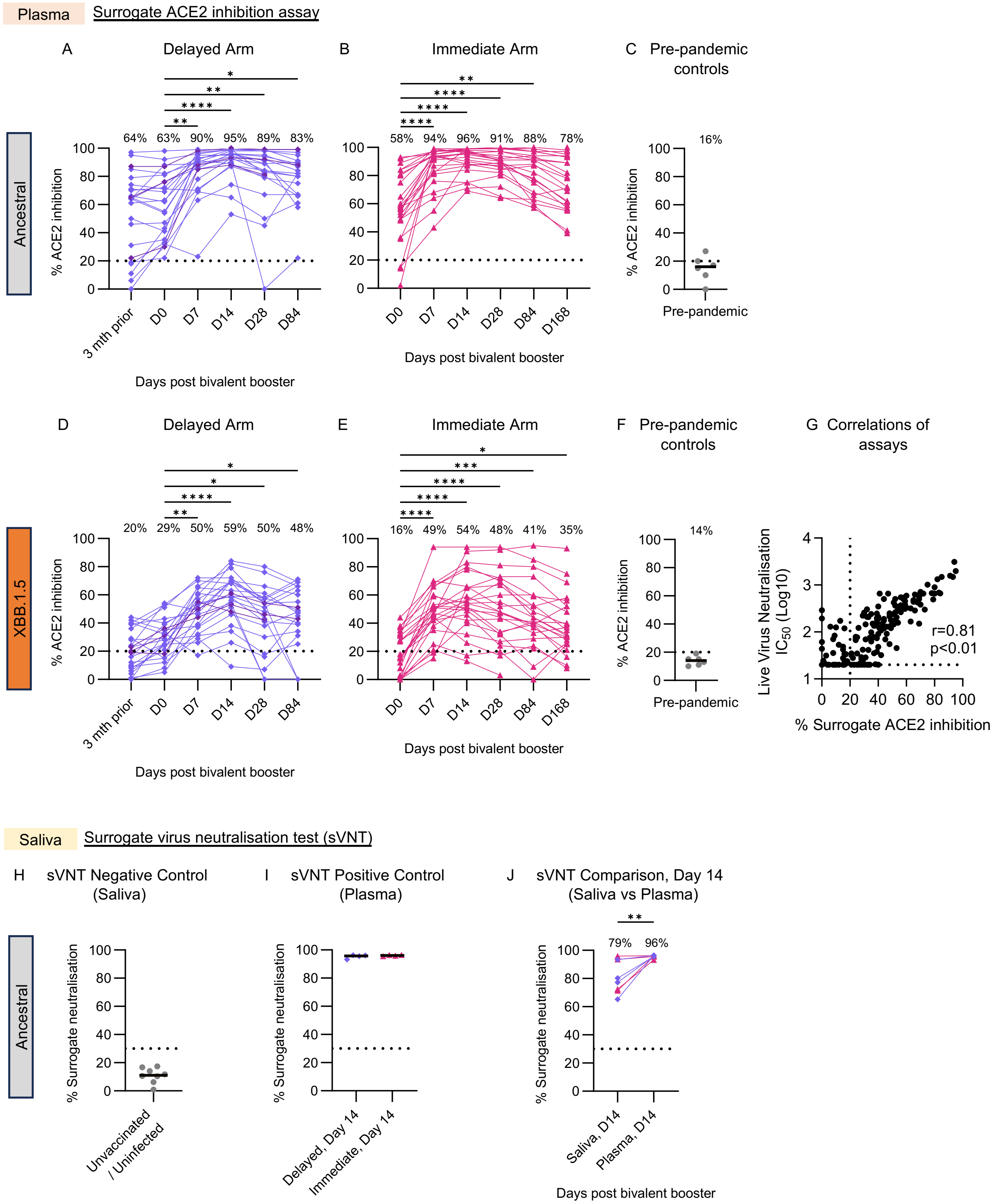

Supplementary Figure 3: Neutralising antibodies using ACE2 inhibition assay.

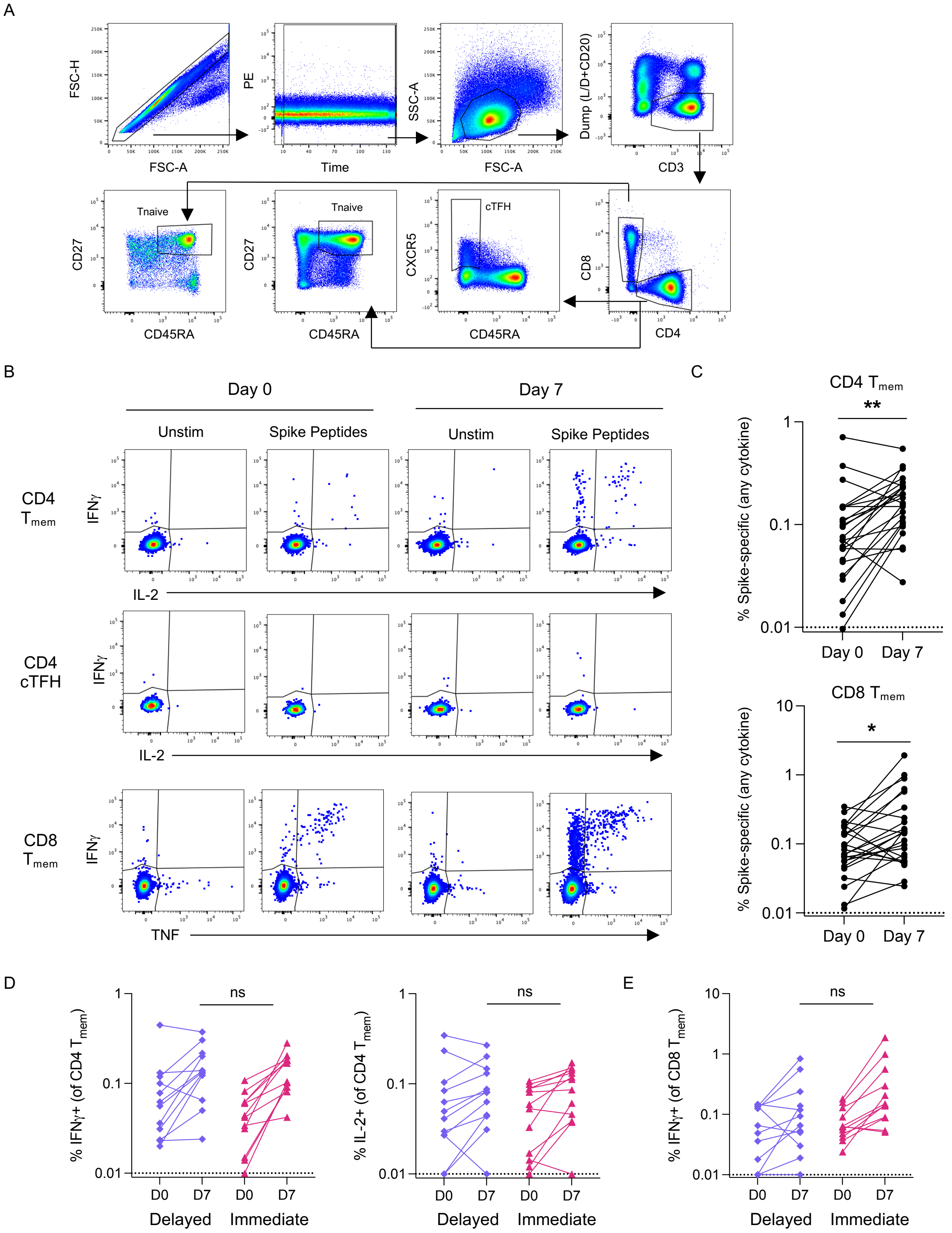

Supplementary Figure 4: Spike-specific CD4 and CD8 T cell responses

Flow cytometry gating strategy for antibody-dependent cellular phagocytosis (ADCP)

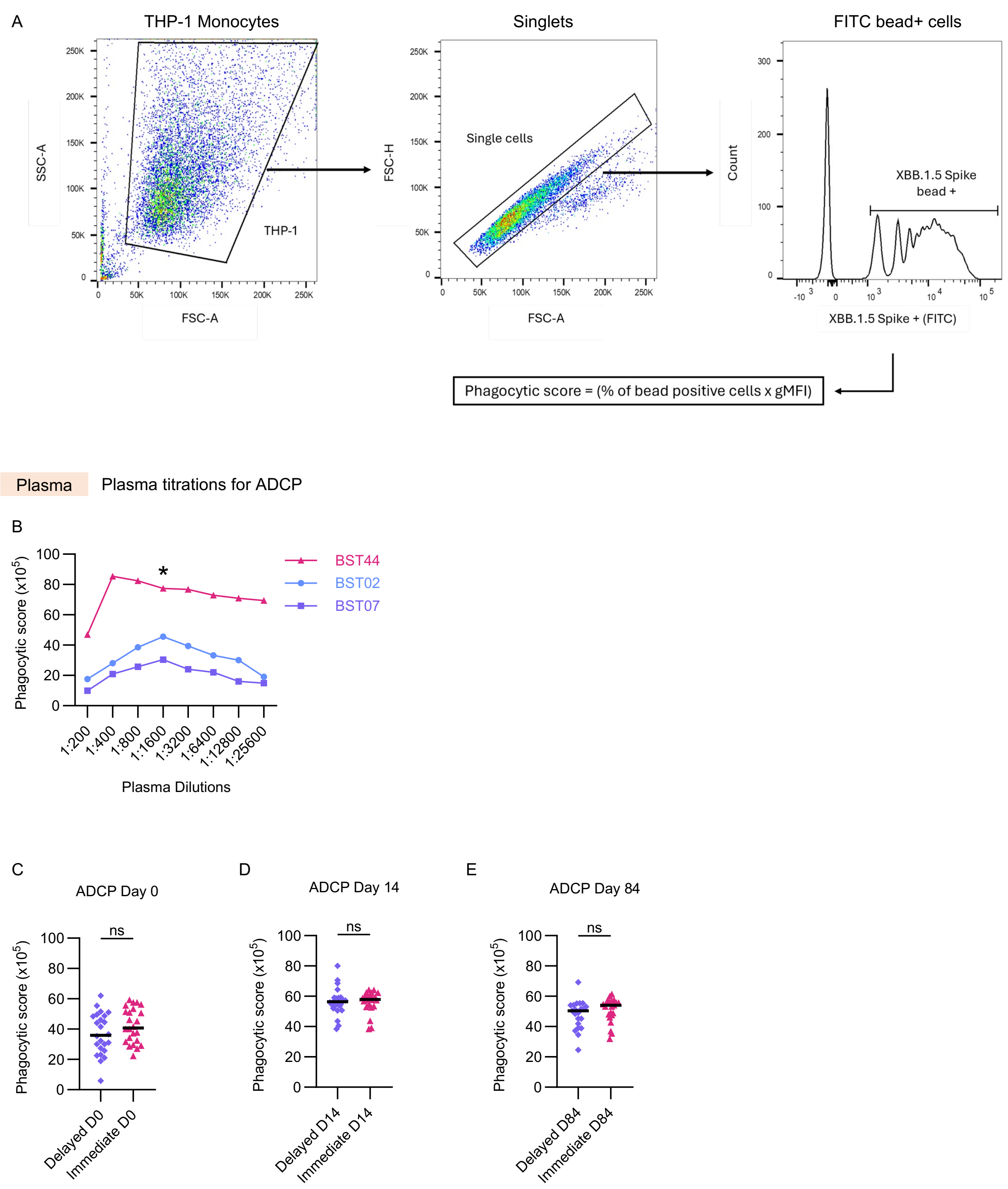

Supplementary Figure 5: Antibody-dependent cellular phagocytosis assay titrations and comparisons.

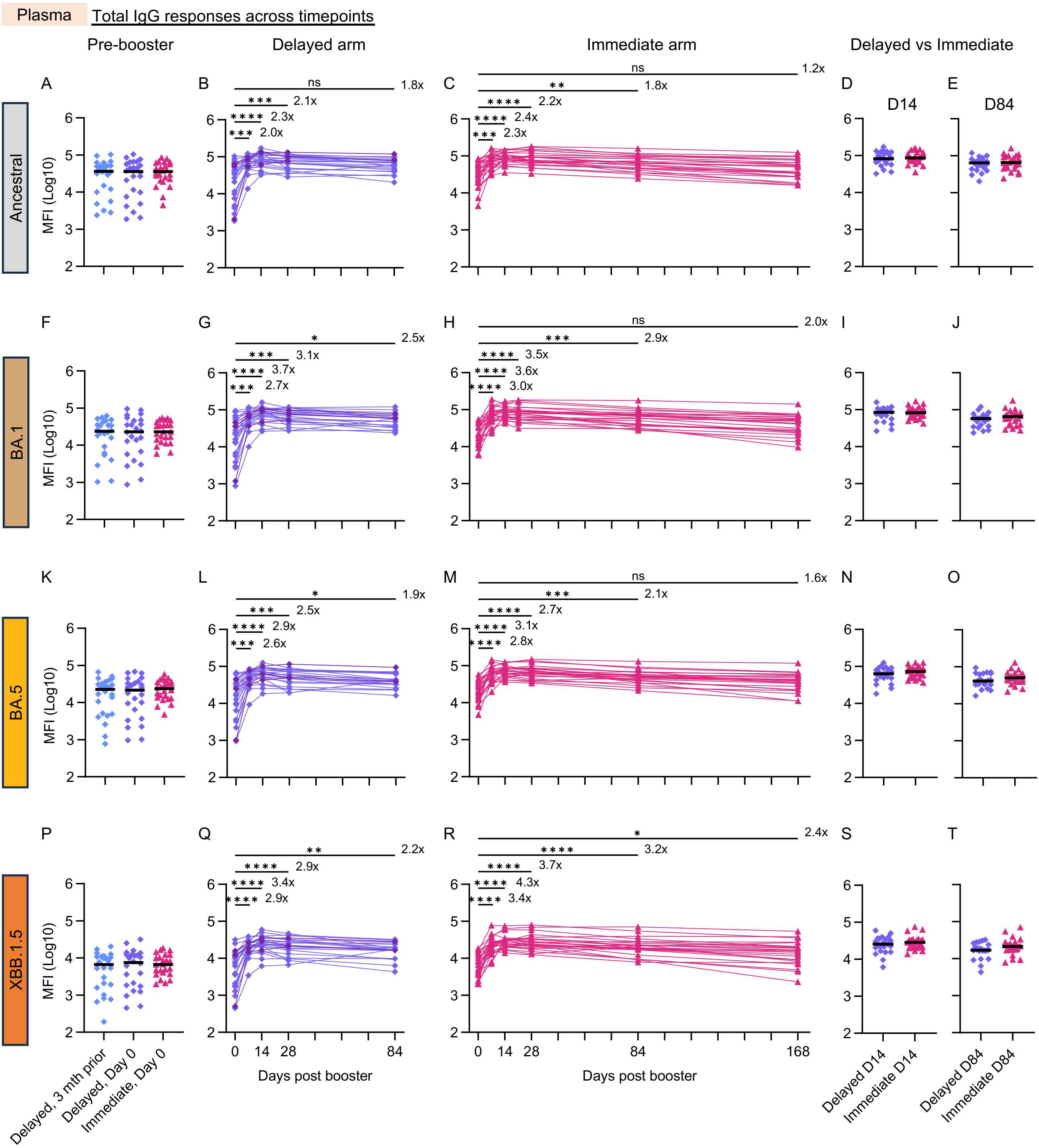

Supplementary Figure 6: Plasma IgG responses following bivalent mRNA booster.

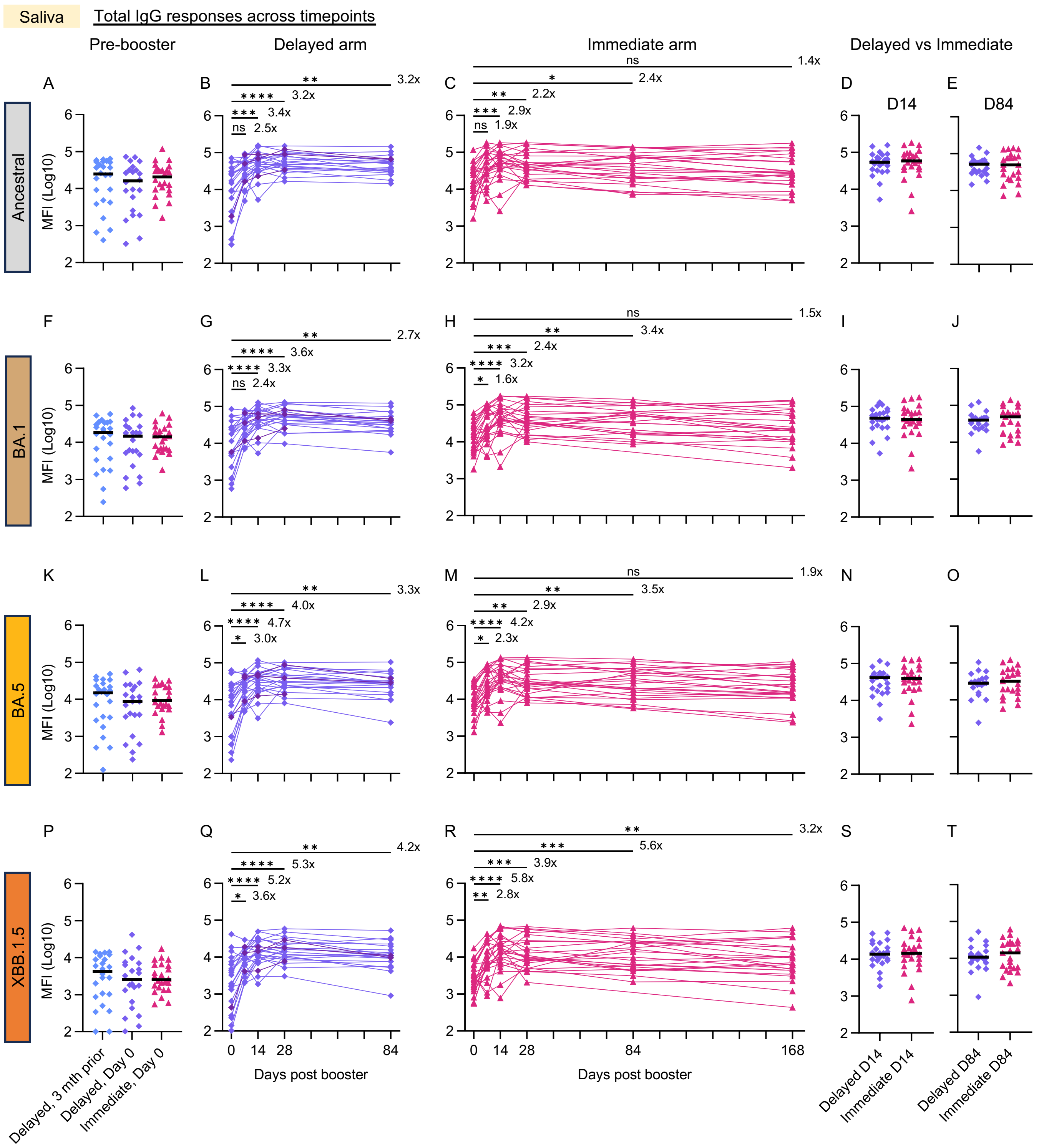

Supplementary Figure 7: Saliva IgG responses following bivalent mRNA booster.

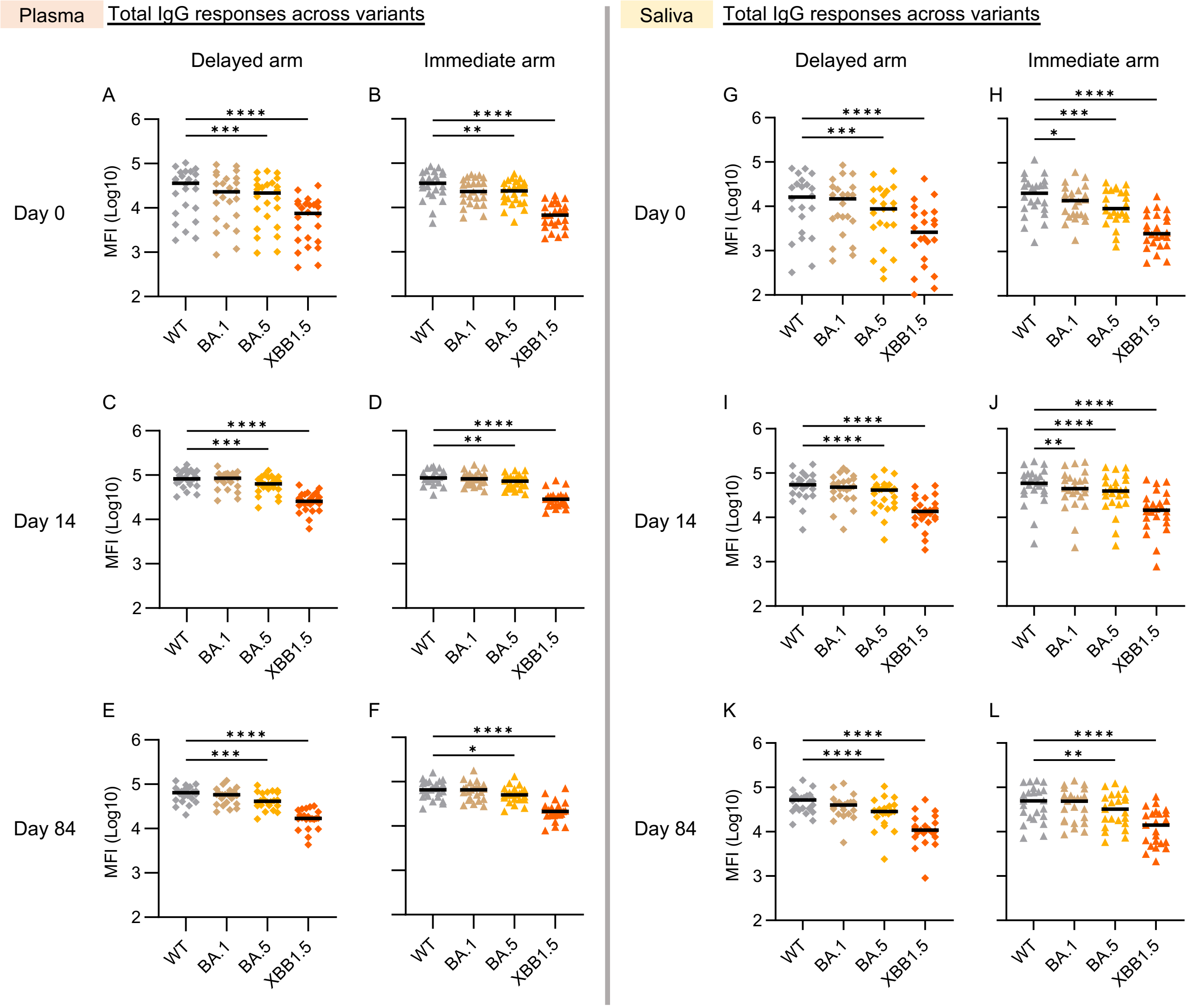

Supplementary Figure 8: Variant IgG responses following bivalent mRNA booster.

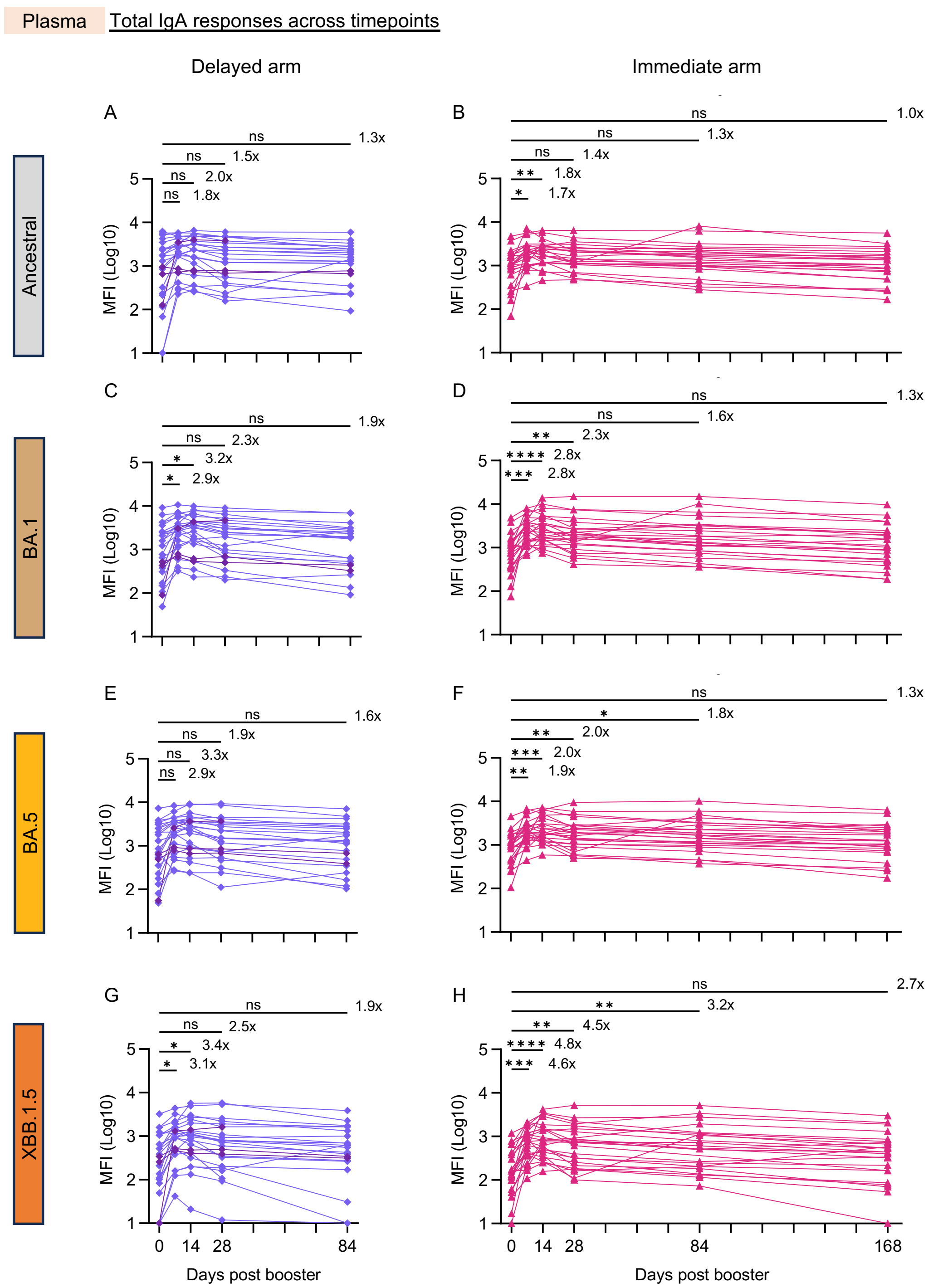

Supplementary Figure 9: Plasma IgA responses following bivalent mRNA booster.

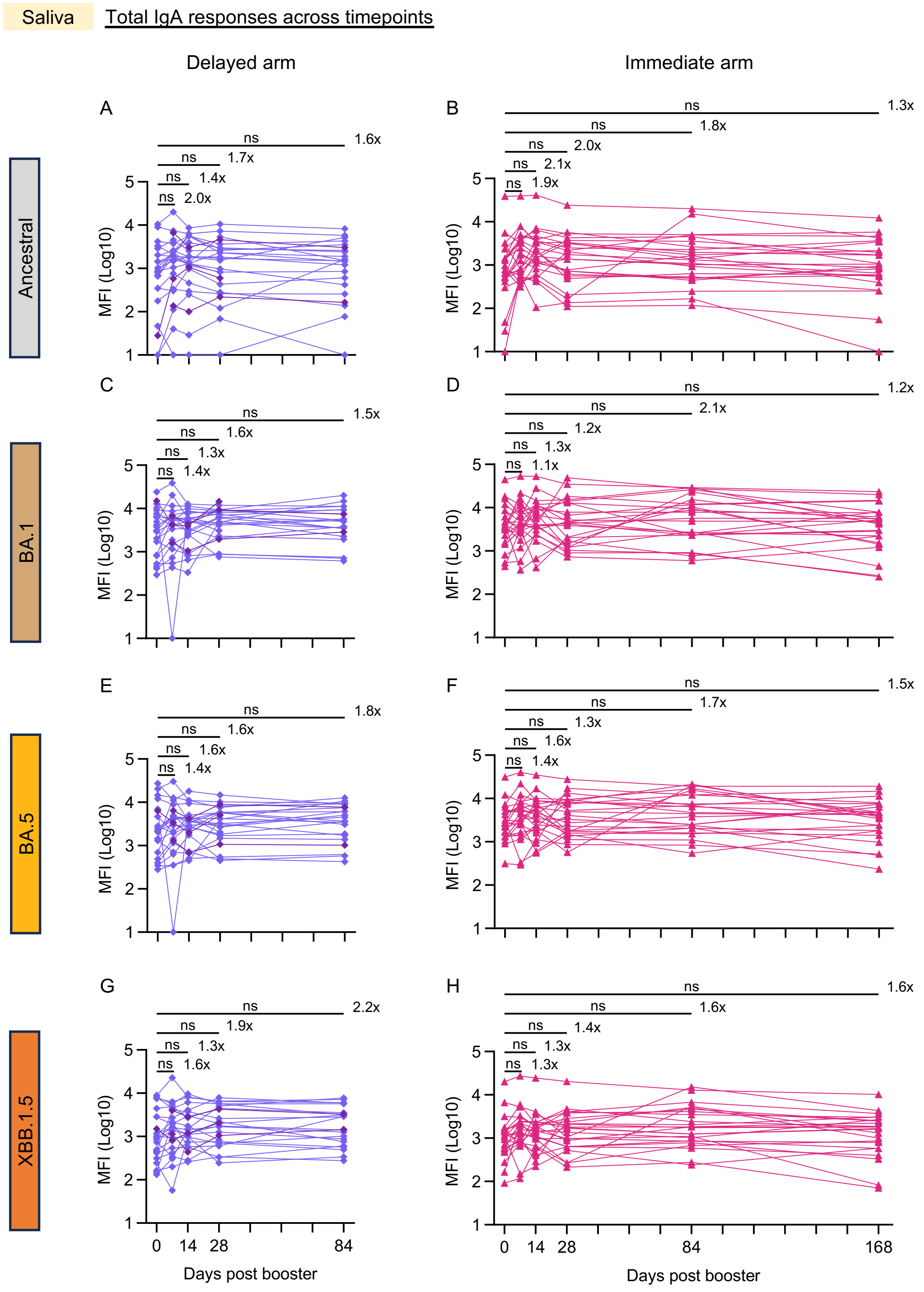

Supplementary Figure 10: Saliva IgA responses following bivalent mRNA booster.

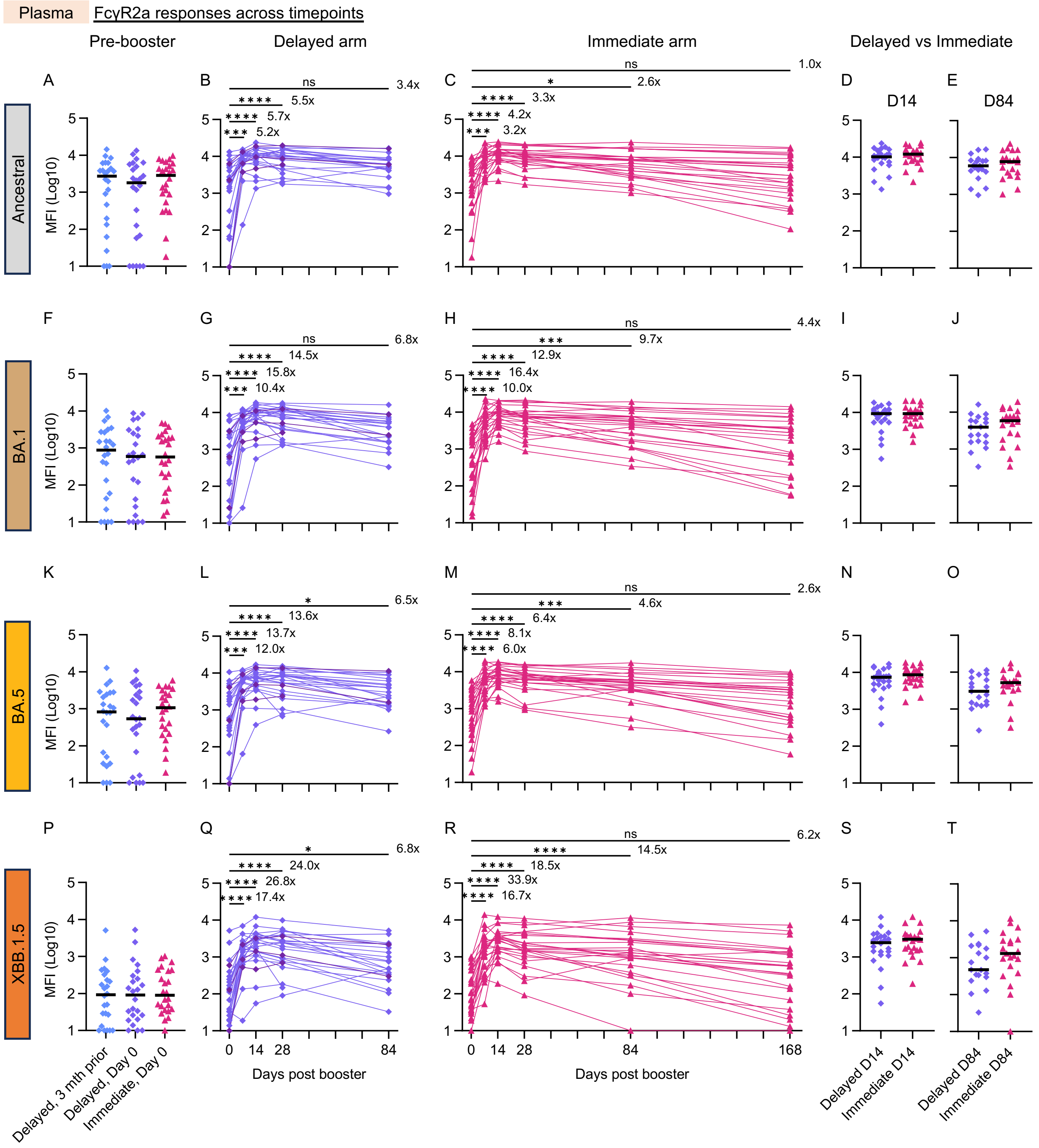

Supplementary Figure 11: Plasma FcγR2a responses following bivalent mRNA booster.

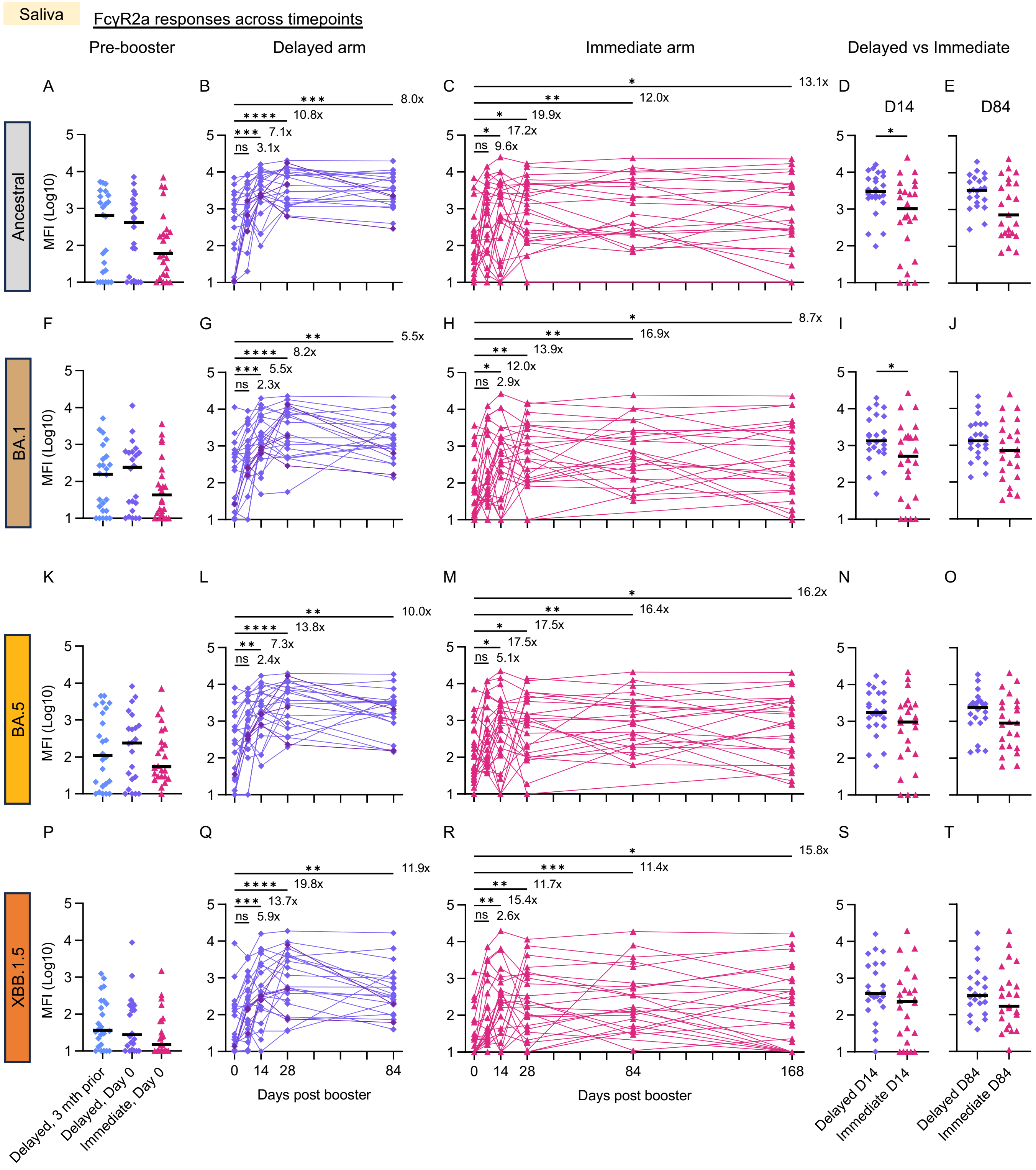

Supplementary Figure 12: Saliva FcγR2a responses following bivalent mRNA booster.

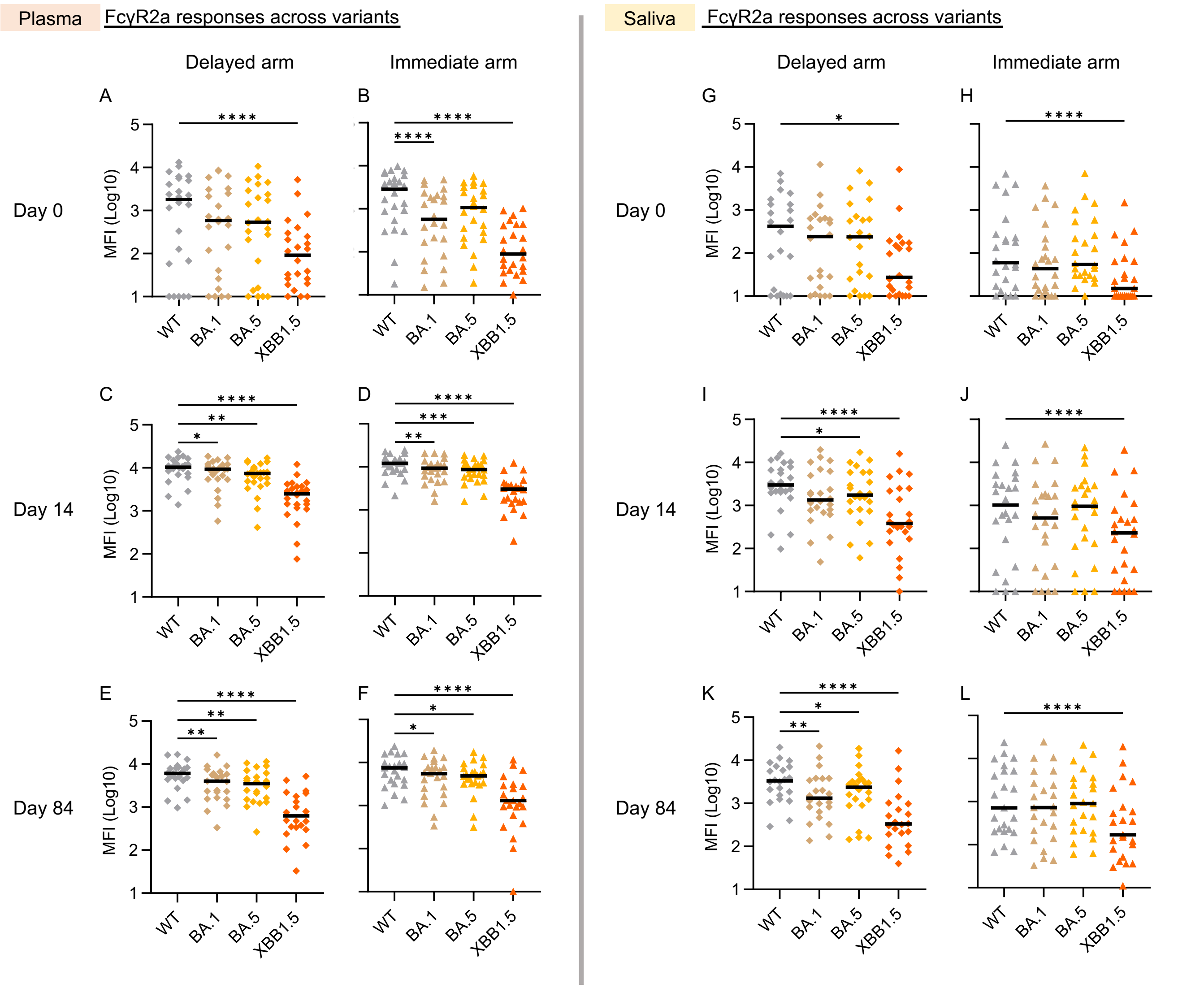

Supplementary Figure 13: Variant FcγR2a responses following bivalent mRNA booster.

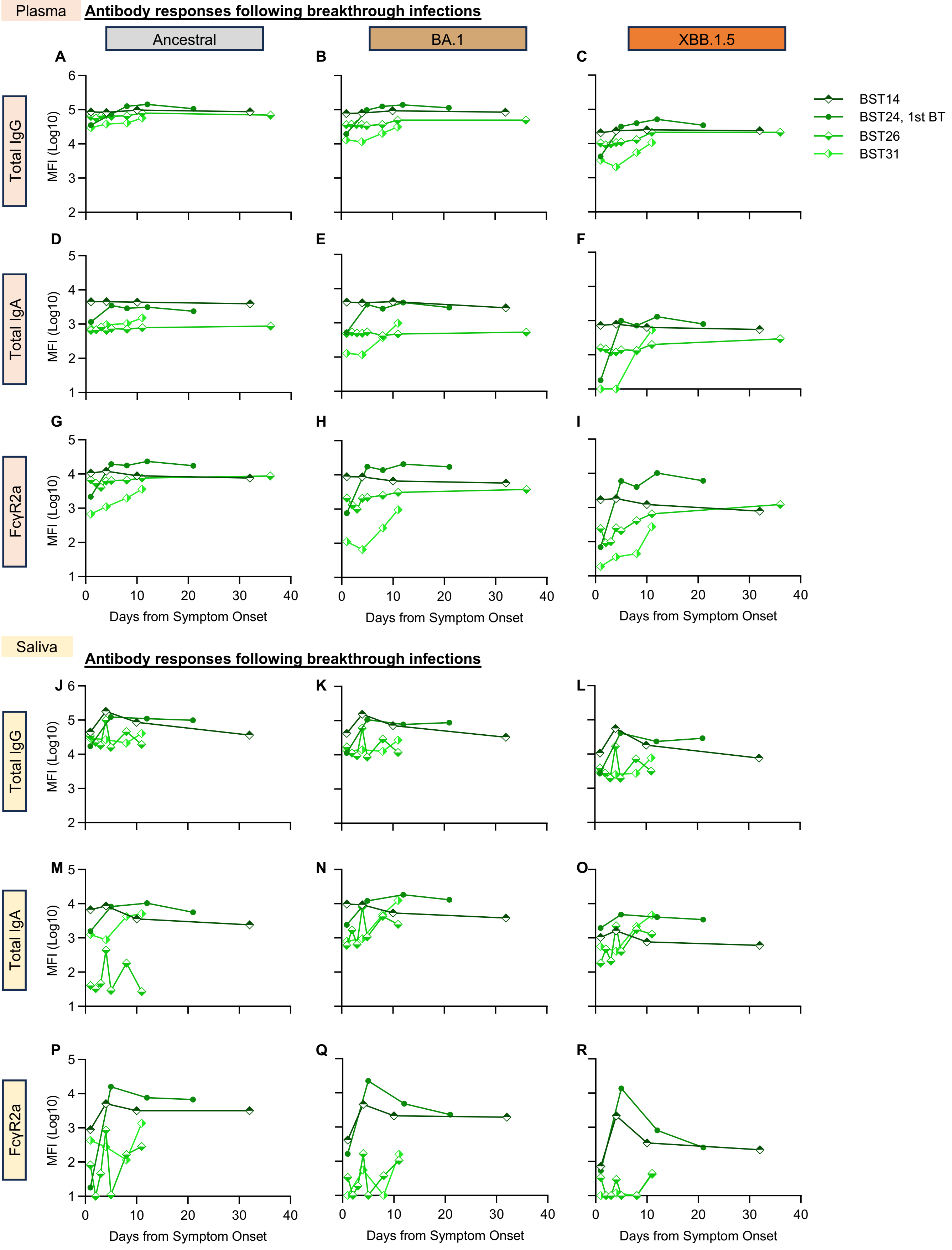

Supplementary Figure 14: Breakthrough COVID-19 variant antibody responses.

Supplemental Table 1: Participant demographics (n=49)

Characteristic	Total received allocation (n=49)	Immediate arm, received allocation (n=25)	Delayed arm, received allocation (n=24)
On study entry			
Age (years)	39.0 (23.6, 62.1)	40.9 (25.2, 62.1)	38.6 (23.6, 61.0)
Sex (M/F), %	46.9/53.1	44.0/56.0	50.0/50.0
Any prior COVID-19 infections (Y/N), %	69.4/30.6	76.0/24.0	62.5/37.5
Number of prior COVID-19 infections	1.0 (1.0, 2.0)	1.0 (1.0, 1.0)	1.0 (1.0, 2.0)
Duration (months) since last COVID-19 infection	6.9 (4.0, 21.3), n=34	6.8 (4.0, 20.5), n=19	7.2 (4.5, 21.3), n=15
Number of previous COVID-19 vaccine doses	3.0 (2.0, 3.0)	3.0 (2.0, 3.0)	3.0 (3.0, 3.0)
On study vaccination †			
Duration (months) since last COVID-19 vaccination	12.9 (9.2, 26.2)	11.4 (9.2, 14.6)	14.5 (9.4, 26.2)
Duration (months) since most recent exposure to COVID-19 spike protein (vaccination or infection)	9.4 (3.7, 18.2)	8.0 (4.2, 13.8)	10.5 (3.7, 18.2)

All data presented as median (minimum, maximum) unless otherwise stated
† Delayed arm participants were vaccinated 12 weeks after enrolment

Supplemental Table 2: Reported adverse events at day 3 and day 7 post-vaccination

	**Reported adverse reactions**	
**Reaction category**	**D3**	**D7**
†Any local reactions	36	2
Pain	35	2
Redness	5	-
Swelling	6	-
†Any systemic reactions	26	-
Headache	10	-
Muscle pain	13	-
Joint pain	7	-
Fatigue	18	-
Fever	5	-
Potential symptoms of myo/pericarditis	1*	-
TOTAL	100	2

†Subjects may report multiple symptoms in each reaction category
*shortness of breath

Supplemental Table 3: Spike-specific CD4 and CD8 T cell responses.

Subset	Cytokine(s)	Median (IQR) Immediate, Day 7	Median (IQR) Delay, Day 7	P-value*
CD4 T_{mem}	IFNg (total)	0.136 (0.079, 0.213)	0.136 (0.091, 0.184)	0.799
	TNF (total)	0.071 (0.029, 0.151)	0.098 (0.038, 0.129)	0.766
	IL-2 (total)	0.081 (0.044, 0.143)	0.099 (0.040, 0.140)	0.946
	IFNg+TNF+IL-2+	0.035 (0.013, 0.075)	0.042 (0.021, 0.076)	0.852
	IFNg+TNF+IL-2-	0.028 (0.013, 0.044)	0.024 (0.015, 0.033)	0.560
	IFNg+TNF-IL-2+	0.020 (0.014, 0.025)	0.019 (0.015, 0.031)	0.670
	IFNg-TNF+IL-2+	0.01 (0.01, 0.019)	0.013 (0.01, 0.021)	0.660
CD8 T_{mem}	IFNg (total)	0.079 (0.035, 0.213)	0.146 (0.089, 0.496)	0.092
	TNF (total)	0.041 (0.018, 0.095)	0.060 (0.014, 0.184)	0.512
	IL-2 (total)	0.017 (0.01, 0.039)	0.026 (0.012, 0.069)	0.352
	IFNg+TNF+IL-2+	0.01 (0.01, 0.020)	0.012 (0.01, 0.027)	0.395
	IFNg+TNF+IL-2-	0.024 (0.012, 0.079)	0.036 (0.017, 0.113)	0.339
	IFNg+TNF-IL-2+	0.01 (0.01, 0.014)	0.012 (0.01, 0.036)	0.237
	IFNg-TNF+IL-2+	0.01 (0.01, 0.01)	0.01 (0.01, 0.01)	N/A

*Statistics assessed by Mann-Whitney *U* test

Supplemental Table 4: On study COVID-19 infections

**COVID-19 infection type**	**Total received allocation (n=49)**	**Immediate arm, received allocation (n=25)**	**Delayed arm, received allocation (n=24)**
Self-reported COVID-19 infection while waiting for study vaccination	3	0	3
Self-reported COVID-19 infection after study vaccination *	11	5	6
Asymptomatic, research laboratory detected COVID-19 infections †, #	10	7	3
**Total** post-randomisation COVID-19 infections	24	12	12

* Includes two participants (one in each study arm) with two COVID-19 infections after study vaccination
† Difference in responses between timepoints that were >4-fold rise in N IgG MFI and a rise in XBB1.5 neutralisation (multiplex bead-based plasma ACE2 inhibition)
# Includes one participant in the delayed arm with an asymptomatic infection detected 9 months before a self-reported infection
